# Associations of adverse childhood experiences with educational attainment and adolescent health and the role of family and socioeconomic factors: A prospective cohort study in the UK

**DOI:** 10.1371/journal.pmed.1003031

**Published:** 2020-03-02

**Authors:** Lotte C. Houtepen, Jon Heron, Matthew J. Suderman, Abigail Fraser, Catherine R. Chittleborough, Laura D. Howe

**Affiliations:** 1 MRC Integrative Epidemiology Unit at the University of Bristol, Population Health Sciences, Bristol Medical School, University of Bristol, Bristol, United Kingdom; 2 School of Public Health, Faculty of Health and Medical Sciences, and Robinson Research Institute, University of Adelaide, Adelaide, Australia; Stellenbosch University, SOUTH AFRICA

## Abstract

**Background:**

Experiencing multiple adverse childhood experiences (ACEs) is a risk factor for many adverse outcomes. We explore associations of ACEs with educational attainment and adolescent health and the role of family and socioeconomic factors in these associations.

**Methods and findings:**

Using data from the Avon Longitudinal Study of Parents and Children (ALSPAC), a prospective cohort of children born in southwest England in 1991–1992, we assess associations of ACEs between birth and 16 years (sexual, physical, or emotional abuse; emotional neglect; parental substance abuse; parental mental illness or suicide attempt; violence between parents; parental separation; bullying; and parental criminal conviction, with data collected on multiple occasions between birth and age 16) with educational attainment at 16 years (*n* = 9,959) and health at age 17 years (depression, obesity, harmful alcohol use, smoking, and illicit drug use; *n* = 4,917). We explore the extent to which associations are robust to adjustment for family and socioeconomic factors (home ownership, mother and partner’s highest educational qualification, household social class, parity, child’s ethnicity, mother’s age, mother’s marital status, mother’s depression score at 18 and 32 weeks gestation, and mother’s partner’s depression score at 18 weeks gestation) and whether associations differ according to socioeconomic factors, and we estimate the proportion of adverse educational and health outcomes attributable to ACEs or family or socioeconomic measures. Among the 9,959 participants (49.5% female) included in analysis of educational outcomes, 84% reported at least one ACE, 24% reported 4 or more ACEs, and 54.5% received 5 or more General Certificates of Secondary Education (GCSEs) at grade C or above, including English and Maths. Among the 4,917 participants (50.1% female) included in analysis of health outcomes, 7.3% were obese, 8.7% had depression, 19.5% reported smoking, 16.1% reported drug use, and 10.9% reported harmful alcohol use. There were associations of ACEs with lower educational attainment and higher risk of depression, drug use, and smoking. For example, odds ratios (ORs) for 4+ ACEs compared with no ACEs after adjustment for confounders were depression, 2.4 (1.6–3.8, *p* < 0.001); drug use, 3.1 (2.1–4.4, *p* < 0.001); and smoking, 2.3 (1.7–3.1, *p* < 0.001). Associations with educational attainment attenuated after adjustment but remained strong; for example, the OR after adjustment for confounders for low educational attainment comparing 4+ ACEs with no ACEs was 2.0 (1.7–2.4, *p* < 0.001). Associations with depression, drug use, and smoking were not altered by adjustment. Associations of ACEs with harmful alcohol use and obesity were weak. For example, ORs for 4+ ACEs compared with no ACEs after adjustment for confounders were harmful alcohol use, 1.4 (0.9–2.0, *p* = 0.10) and obesity, 1.4 (0.9–2.2, *p* = 0.13) We found no evidence that socioeconomic factors modified the associations of ACEs with educational or health outcomes. Population attributable fractions (PAFs) for the adverse educational and health outcomes range from 5%–15% for 4+ ACEs and 1%–19% for low maternal education. Using data from multiple questionnaires across a long period of time enabled us to capture a detailed picture of the cohort members’ experience of ACEs; however, a limitation of our study is that this resulted in a high proportion of missing data, and our analyses assume data are missing at random.

**Conclusions:**

This study demonstrates associations between ACEs and lower educational attainment and higher risks of depression, drug use, and smoking that remain after adjustment for family and socioeconomic factors. The low PAFs for both ACEs and socioeconomic factors imply that interventions that focus solely on ACEs or solely on socioeconomic deprivation, whilst beneficial, would miss most cases of adverse educational and health outcomes. This interpretation suggests that intervention strategies should target a wide range of relevant factors, including ACEs, socioeconomic deprivation, parental substance use, and mental health.

## Abstract 2

In a prospective cohort study, Houtepen and colleagues investigate associations between adverse childhood experiences, educational and health outcomes, and socioeconomic and family factors in children in the UK.

## Author summary

**Why was this study done?:**

- Adverse childhood experiences (ACEs) are associated with lower educational attainment and worse health.
- However, the role of family and socioeconomic factors in these associations—which could act both as confounders of the associations or modify the effects of ACEs—is underexplored, as are the relative contributions of ACEs and family and socioeconomic factors to the outcomes.

**What did the researchers do and find?:**

- We used data from a prospective cohort study to quantify associations between exposure to ACEs and educational and health outcomes in adolescence.
- We assessed whether associations were robust to adjustment for family and socioeconomic factors and whether associations between ACEs and outcomes differed by socioeconomic position.
- We also estimate the proportion of adverse educational and health outcomes attributable to ACEs or family or socioeconomic measures.

**What do these findings mean?:**

- People who experience ACEs have, on average, lower educational attainment and higher risk of depression, drug use, and smoking; these patterns were not fully explained by family and socioeconomic factors.
- We did not find strong evidence of associations between ACE and obesity or alcohol use.
- Our findings imply that interventions that focus solely on ACEs or solely on socioeconomic deprivation, whilst beneficial, would miss most cases of adverse educational and health outcomes, suggesting that intervention strategies should target a wide range of relevant factors, including ACEs, socioeconomic deprivation, parental substance use, and mental health.

## Introduction

There is increasing awareness of the role that adverse childhood experiences (ACEs) can play in influencing educational attainment, physical and mental health [1–6]. For example, in the Environmental Risk (E-Risk) Longitudinal Twin Study, child maltreatment was associated with a doubling in the odds of having low educational qualifications or of not being in education, employment, or training at age 18 years [1]. ACEs are rising rapidly on policy agendas, and there is a drive within public health and education to prevent ACEs, develop and implement interventions to improve resilience, and promote ACE-aware services [7–10].

The definition of ACEs varies between studies and is the subject of debate [11], but the adversities most commonly studied include child maltreatment (for example, emotional, physical, and sexual abuse; physical or emotional neglect) and measures of household dysfunction (for example, violence between parents, parental separation and parental substance misuse, mental illness or criminal behaviour). In the Avon Longitudinal Study of Parents and Children (ALSPAC), a birth cohort study that recruited pregnant women in the southwest of England between 1991–1992, 21% of participants have experienced 4 or more ACEs [12]. It is well established that these adverse experiences are not randomly distributed across a population; socioeconomic disadvantage is a strong risk factor for ACEs [13–15]. In ALSPAC, we have previously shown that people from families with low social class (based on parental occupation) have twice the prevalence of 4 or more ACEs compared with young people from high social class families. There is some debate about whether poverty should itself be considered an ACE; advocates point to the link between poverty and ACEs and the advocacy advantages of having poverty included in the definition of ACEs [16], whereas detractors view poverty as a structural issue and highlight that ACEs occur across the socioeconomic spectrum [17]. Here, we view socioeconomic disadvantage and ACEs as separate phenomena. Our conceptual framework posits that socioeconomic factors can potentially both cause ACEs (and therefore act as confounders of the relationships between ACEs and subsequent outcomes) and modify the effects of ACEs. For example, high socioeconomic position may provide resources (personal, material, and social) that could act to mitigate against some of the adverse consequences of ACEs.

Despite socioeconomic disadvantage being a major risk factor for ACEs, the conversation about ACEs rarely focuses on the interrelationships between ACEs and socioeconomic conditions or other family-level factors such as maternal age or smoking and the implications of this for policy [18]. For example, studies vary in the degree to which they adjust for potential confounding by socioeconomic conditions or family-level factors, and whilst there is an extensive literature on factors that promote resilience to ACEs (particularly maltreatment) [19–21], there is relatively little focus in the literature on whether associations between ACEs and adverse outcomes are weaker in children from socioeconomically advantaged families (one way of conceptualising resilience).

In this paper, we use data from a United Kingdom prospective cohort study to examine the associations of ACEs from 0 to 16 years with educational attainment at 16 years (end of compulsory education) and markers of adolescent health and health-related behaviours assessed at age 17 years (depression, obesity, harmful alcohol use, smoking, and illicit drug use). We assess the degree to which these associations are robust to adjustment for a wide range of family and socioeconomic characteristics, and we test the hypothesis that associations between ACEs and education and health outcomes will be stronger in people from families with low socioeconomic position. We calculate population attributable fractions (PAFs) for each outcome for ACEs and for several socioeconomic and family-related measures to assess the relative contributions of each of these factors to adverse educational and health outcomes, with the motivation of understanding the proportion of cases of these adverse outcomes that could potentially be prevented by interventions focused solely on ACEs.

## Methods

### Participants

ALSPAC is a prospective, population-based birth cohort study that recruited 14,541 pregnant women resident in Avon, UK, with expected delivery dates between 1st April 1991 and 31st December 1992 [22, 23]. When the oldest children were approximately 7 years of age, an attempt was made to bolster the initial sample with eligible cases who had failed to join the study originally. The total sample size for analyses using any data collected after the age of 7 is therefore 15,454 pregnancies, resulting in 15,589 foetuses. Of these, 14,901 were alive at 1 year of age. The mothers, their partners, and the child have been followed up using clinics, questionnaires, and links to routine data. The study website contains details of all the data that are available through a fully searchable data dictionary: http://www.bristol.ac.uk/alspac/researchers/our-data/. Ethical approval for this study was obtained from the ALSPAC Law and Ethics Committee and the Local Research Ethics Committees, and participants provided written informed consent. This study uses data from pregnancy through to a questionnaire administered to the participants at age 22 years.

We excluded children with data on fewer than 10% of the ACE questions across all time points (*n* = 2,604). This threshold was used to ensure sufficient data to inform multiple imputation whilst minimising the number of people excluded from analyses because of missing data, particularly because missingness was related to both exposure to ACEs and experience of the outcomes of interest. After this exclusion, 11,935 participants remained. We created separate analysis samples for analysis of educational attainment and health outcomes by restricting these to participants with at least one outcome measure; we also excluded one child from within each twin pair at random (*n* = 152) to maintain independence of observations. This resulted in final sample sizes of 9,959 for educational outcomes (obtained through linkage to routine data) and 4,917 for health outcomes (assessed at a research clinic).

### ACEs

Data on multiple forms of ACEs were reported by both participants themselves and their mothers at multiple time points, primarily through questionnaires. We used data from 582 questions relevant to one or more ACEs. The majority of the early-life ACE data (0–8 years) are parent reported, but when the children were 8 years old, they began self-reporting ACEs. Additionally, in their twenties, the participants retrospectively reported on child maltreatment (several forms of abuse and neglect) as well as whether their parents were violent towards each other. The majority of ACE data were collected prospectively, but we also included retrospective self-report measures because these complement the prospective data. For instance, the sexual abuse rates prospectively reported by parents were much lower than those retrospectively self-reported by the participants. Full details of the derivation of ACE measures, including the exact phrasing of questions, definitions, and ages at which data were collected, have been described previously [12]. Briefly, dichotomous constructs indicating exposure to adversities between birth and 16 years were created for the 10 ACEs that are included in the World Health Organization ACE international questionnaire [24]:

1. ever sexually abused or forced to perform sexual acts or touch someone in a sexual way (sexual abuse)
2. adult in family was ever physically cruel towards or hurt the child (physical abuse)
3. parent was ever emotionally cruel towards the child or often said hurtful/insulting things to the child (emotional abuse)
4. child always felt excluded, misunderstood, or never important to family, parents never asked or never listened when child talked about their free time (emotional neglect)
5. parent was a daily cannabis or any hard drug user or had an alcohol problem (parental substance abuse)
6. parent was ever diagnosed with schizophrenia or hospitalised for a psychiatric problem or, during the first 18 years of the child’s life, parent had an eating disorder (bulimia or anorexia), used medication for depression or anxiety, attempted suicide, or scored above previously established cutoffs for depression (Edinburgh Postnatal Depression Scale (EPDS) >12–13) (parental mental illness or suicide)
7. parents were ever affected by physically cruel behaviour by partner or ever violent towards each other, including hitting, choking, strangling, beating, and shoving (violence between parents)
8. parents separated or divorced (parental separation)
9. child was a victim of bullying on a weekly basis (bullying)
10. parent was convicted of a crime (parental criminal conviction)

Multiple questions feed into each ACE using predetermined criteria; an ACE is assumed to have been experienced if it is reported in one or more of the questions, even if there are inconsistencies between the questions. The definitions are described in S1 Table and S1 Text. S2 Table contains a list of the questionnaires used.

### Educational attainment

ALSPAC data were linked to the National Pupil Database (NPD). This is a governmental database providing data on pupil level attainment in state-funded schools in England. General Certificate of Secondary Education (GCSE) examinations are sat during the 11th year of compulsory schooling when children are aged 15–16 (years 2007–2009 for the ALSPAC cohort). Pupils study up to 12 subjects (8 on average). The subjects are graded individually on a scale of A* (highest) to G (lowest). For this analysis, we used a dichotomous indicator of fewer than 5 GCSEs at grades A*–C including English and Mathematics, which is a widely used benchmark of academic achievement in the UK and a requirement for entry into many further education courses. As an additional outcome, following a suggestion from a reviewer, we used a continuous score of ‘GCSE points’; this is the total score of an individual’s top 8 qualifications ranked in terms of points. The capped score is more suitable than the total score because the number of GCSE qualifications taken by a pupil varies, but the majority take at least 8.

### Health and health-related behaviours at age 17

During a research clinic at age 17 years (mean 17 years and 9 months, SD 4 months), weight was measured to the nearest 50 g using the Tanita Body Fat Analyzer (Model TBF 401A; Tanita Corporation, Amsterdam, the Netherlands), with participants in underwear or light clothing and footwear removed. Height was measured using a Harpenden stadiometer to the last complete mm, with participants unshod. Body mass index (BMI) was calculated as weight in kilograms divided by height in metres squared. Substance use and mental health were assessed using self-administered computer-assisted interviews. We derived the following dichotomous indicators:

- Obesity: BMI was converted into sex- and age-specific Z-scores relative to UK 1990 population reference data. These Z-scores were used to define obesity based on published BMI Z-score cutoffs from the International Obesity Task Force (BMI Z ⩾ 2.212 for boys and BMI Z ⩾ 2.195 for girls).
- Regular smoking: we created a dichotomous indicator of smoking weekly or more versus no smoking or smoking less than weekly.
- Harmful drinking: ⩾16 on the 10-item alcohol use disorders identification test (AUDIT) [25].
- Depression: based on the clinical interview schedule—revised (CIS-R), defined as meeting the depression diagnosis criteria of the international classification of diseases, 10th revision [26].
- Illicit use of drugs: problematic cannabis use or, in the past 12 months, any use of any of the following substances: cocaine, amphetamines, inhalants, sedatives, hallucinogens, or opioids. Problematic cannabis use was measured using the six-item cannabis abuse screen test [27], which assesses cannabis consumption in the previous 12 months and focuses on difficulties controlling use and associated health and social impairment. All items are answered on a 5-point scale (0 never, 1 rarely, 2 from time to time, 3 fairly often, and 4 very often). A response of fairly often or very often to any of the 6 items was used to indicate problem cannabis use.

Additionally, following a suggestion from a reviewer, we used BMI as a continuous variable.

### Confounders

At enrolment and prior to delivery, several self-report questionnaires were administered that measured socioeconomic, family, and (mental) health variables. Based on these parental questionnaires, the following covariables were included in the analysis: mother’s home ownership status during pregnancy (mortgaged/owned/council rented/furnished private rental/unfurnished private rental/housing authority rented/other), mother and partner’s highest educational qualification (CSE/vocational/O-level/A-level/degree), household social class (highest of mother and partner social class according to the Registrar General’s Social Classes: professional/managerial and technical/skilled nonmanual/partly skilled/unskilled), parity, maternal report of child’s ethnicity (white/nonwhite), mother’s age at delivery (in years), mother’s marital status during pregnancy (never married/widowed/divorced/separated/first marriage/marriage 2 or 3), mother’s depression score (EPDS) at 18 and 32 weeks gestation, and mother’s partner depression score (EPDS) at 18 weeks gestation. The variables were chosen by searching the ALSPAC variable catalogue for variables related to key concepts determined a priori. The first and last author reviewed the list and decided on a set of variables we felt captured all relevant concepts, using what we felt to be the best measure of each based on the level of missing data and likely degree of measurement error. More details on these variables and their distributions are available in S3 Table.

### Missing data

Because of the derivation of ACE measures from multiple questionnaires and clinics over a long time period (birth–23 years), no participants had data on all of the individual questionnaire items, necessitating the use of multivariate multiple imputation. Ideally, we would impute missing values of each questionnaire item, but the lack of complete cases in combination with the high number of variables (>500 separate questions relating to ACEs) led to convergence errors. Therefore, we adopted a pragmatic approach to imputation, adapted from the scale-level imputation method proposed by Enders [28]. We derived a dichotomous construct indicating presence or absence of each ACE. If a participant responded to 50% or more of the questions related to a given ACE, we used these data to create the dichotomous indicator. If the participant responded to less than 50% of the questions, we set the dichotomous indicator to missing. We derived a cumulative adversity measure (ACE score) by summing exposure to the 10 classic ACEs, defining 4 categories (0, 1, 2–3, and more than 4 ACEs). Because of the considerably larger sample size available for educational attainment than for health measures, these groups of outcomes were considered in separate imputation models. Because there are some sex differences in ACE prevalence (S4 Table) and potentially higher-order interactions between sex and adversity that we want to preserve, males (education *n* = 5,023, health *n* = 2,163) and females (education *n* = 4,936, health *n* = 2,754) were imputed separately before appending the two data sets before analysis. The dichotomous ACE indicators and the ACE score were included in multiple imputation models, along with all outcome variables, all covariates detailed above, and additional auxiliary variables likely to predict missingness, ACE exposure, or health status (sociodemographic indicators, adversity measures from before the child’s birth, and additional education and health variables; additional details in S5 Table and full details in previous publication [12]). For both males and females, 90 imputed data sets were created using the mice package [29] in R version 3.3.1 with 30 iterations per data set. For secondary analyses exploring interactions between ACEs and parental social class or maternal education, imputation models were rerun stratified by dichotomous indictors of i) parental social class (manual versus nonmanual, highest social class of mother or partner) and ii) maternal education (CSE, vocational education, or lower versus O-level, A-level, degree, or higher).

### Statistical analyses

All statistical modelling was done in R version 3.3.1 unless otherwise specified, using binary logistic regression models for all binary outcomes and linear regression for the continuous outcomes of GCSE point score and BMI. Associations of each separate ACE and an ACE score (categorised to give comparability with previous studies) with each outcome were assessed in an unadjusted model and a basic model (adjusted for sex), as well as a fully adjusted model (adjusted for home ownership, maternal and partner education, household social class, parity, ethnicity, maternal age, maternal marital status, and maternal and partner depression during pregnancy). The individual ACEs are analysed separately in order to provide detail on whether certain ACEs are driving any associations of the ACE score or whether the patterns of associations differ across types of ACEs. For the imputed data, the logistic regression results and numerators and denominators for descriptive analyses were obtained by averaging across the results from each of the 90 imputed data sets using Rubin’s rules. This procedure appropriately modifies the standard errors for regression coefficients (used to calculate *p*-values and 95% confidence intervals [CIs]) to take account of uncertainty in both the imputations and the estimate. Likelihood ratio test statistics were combined using an approximation proposed by Meng and Rubin [30]. As a sensitivity analysis, we replicated these analyses in people with ‘complete’ data, i.e., participants who responded to more than 50% of the questionnaire items for all ACEs and who had data on the outcomes.

To examine whether the associations differed according to sex, we used likelihood ratio tests for interaction and, if applicable, report the results of sex-stratified analyses. We also used likelihood ratio tests to assess interactions between ACEs and manual versus nonmanual parental social class and low versus high maternal education.

Prevalence of the outcomes across exposure categories and risk differences (RDs) and ratios were estimated in the imputed data using the ‘mim: glm’ command in Stata version 15. PAFs were estimated using the formula $PAF=\frac{Ppop\;x\;(RR-1)}{Ppop\;x\;(RR-1)+1}x\;100$, where *Ppop* is the proportion of exposed participants and *RR* is the risk ratio. The PAF estimates the percentage of cases of the outcome that would be prevented if the exposure was eliminated (assuming causality and absence of bias). Alternatively, it could be conceptualised as the percentage of people who go on to develop the adverse outcome that would be included in interventions targeted at the risk factor of interest. This analysis was performed for each of our binary outcomes for the following exposures, which were selected to represent a range of potential ways of identifying high-risk groups, most of which have a prevalence broadly similar to 4 or more ACEs (prevalences reported are in the imputed data used for analysis of educational attainment): 4 or more ACEs (19%), low maternal education (CSE, vocational qualifications, or lower; 30%), manual social class (classes IIIm, IV, or V in the 1991 UK Office of Population Censuses and Surveys classification; 23%), maternal depression in pregnancy (score of 12 or more on the EPDS on either of the two pregnancy questionnaires; 21%), any self-reported maternal smoking during pregnancy (26%), social housing during pregnancy (15%), and maternal age less than 20 years (4%).

A brief project plan was submitted to the ALSPAC study executive prior to starting analyses (see S2 Text). The following changes were made after this initial plan was written: i) our initial proposal focused only on educational attainment as an outcome, but we decided to broaden the focus to include health outcomes; ii) we initially proposed to include only socioeconomic factors as potential confounders but subsequently realised that other family-level variables could also be important confounders; and iii) after discussing preliminary results amongst co-authors, we decided to include calculation of PAFs. Following reviewer suggestions, we also added analysis of BMI and educational attainment as continuous variables and described the associations between health outcomes. As an additional sensitivity analysis suggested by a reviewer, we repeated the main analysis recoding maternal age as a categorical variable (less than 20 years, 20–34 years, or over 35 years). A STROBE (STrengthening the Reporting of OBservational studies in Epidemiology) checklist is available in S1 Checklist.

## Results

84% of ALSPAC participants were exposed to at least one ACE; 23.6% were exposed to 1 ACE, 36.5% to 2 or 3 ACEs, and 23.8% to 4 or more ACEs (Table 1; prevalences reported here are for the participants included in the analysis of educational attainment). The distribution of the ACE score was similar in males and females (*p* = 0.32 for the participants included in analysis of educational outcomes; *p* = 0.21 for participants included in analysis of health outcomes; S4 Table). The prevalence of individual ACEs ranged from 4.1% for sexual abuse to 48.6% for parental mental health problems. There was evidence of sex differences in the prevalence of physical abuse (17.1% in males, 20.9% in females; *p* < 0.01), sexual abuse (2.3% in males, 6.0% in females; *p* < 0.01), emotional neglect (26.5% in males, 21.2% in females; *p* < 0.01), and bullying (28.6% in males, 23.7% in females; *p* < 0.01). The prevalence of other ACEs was similar in males and females (S4 Table). Consistent with a higher rate of missing data in participants with lower socioeconomic position, who are more likely to drop out from the cohort [31], the ACE prevalence estimates were higher in the imputed data compared with the raw data [12]; for example, in the observed data, 14.3% of participants in the education analysis sample reported 4 or more ACEs, compared with 23.8% in the imputed data (S3 Table).

**Table 1:** Participant characteristics. Characteristics of the participants included in analyses using data from multivariate multiple imputation. Because the data are from multiple imputation models, numerators and denominators are estimated by averaging across imputed data sets. All prevalences reported here relate to the data sets used for analysis of educational attainment, *N* = 9,959, apart from health outcomes, where *N* = 4,917.

	Percentage (n/N)
ACE score: 0	16.1 (1,605/9,959)
1	23.6 (2,350/9,959)
2–3	36.5 (3,634/9,959)
4+	23.8 (2,370/9,959)
Physical abuse	19.0 (1,888/9,959)
Sexual abuse	4.1 (411/9,959)
Emotional abuse	23.9 (2,378/9,959)
Emotional neglect	23.9 (2,381/9,959)
Bullying	26.2 (2,607/9,959)
Violence between parents	25.3 (1,502/9,959)
Parental substance abuse	15.1 (1,502/9,959)
Parental mental health problems or suicide attempt	48.6 (4,840/9,959)
Parental criminal conviction	10.5 (1,050/9,959)
Parental separation	33.8 (3,371/9,959)
5+ GCSEs including math and English at grades A*–C	54.5 (5,424/9,959)
Obesity at age 17	7.3 (357/4,917)
Depression at age 17	8.7 (426/4,917)
Smoking at age 17	19.5 (958/4,917)
Illicit drug use at age 17	16.1 (791/4,917)
Harmful alcohol use at age 17	10.9 (539/4,917)

5+ GCSEs indicates 5 or more grades A*–C including English and Mathematics from GCSE or equivalent examinations. GCSEs are qualifications obtained at age 16, which was the end of compulsory education for this cohort. Obesity is defined as BMI Z-score above cutoffs from the International Obesity Task Force (BMI Z ⩾ 2.212 for boys and BMI Z ⩾ 2.195 for girls). Depression is defined as meeting the depression diagnosis criteria of the international classification of diseases, 10th revision on the CIS-R. Smoking is defined as weekly smoking. Illicit drug use is defined as problematic cannabis use (based on the 6-item cannabis abuse screen test) or, in the past 12 months, any use of any of the following substances: cocaine, amphetamines, inhalants, sedatives, hallucinogens, or opioids. Harmful alcohol use is defined as ⩾16 on the 10-item AUDIT. **Abbreviations:** ACE, adverse childhood experience; AUDIT, alcohol use disorders identification test; BMI, body mass index; CSI-R, clinical interview schedule—revised; GCSE, General Certificate of Secondary Education.

Just over half (54.5%) of participants received 5 or more GCSEs at grade C or above including English and Maths (Table 1). The prevalence of health outcomes at age 17 years was 7.3% for obesity, 8.7% for depression, 19.5% for smoking, 16.1% for drug use, and 10.9% for harmful alcohol use (Table 1). There were associations between the outcomes; for example, 45% of participants who smoked also reported drug use, and 20% of participants reporting depression also reported harmful alcohol use (S6 Table).

There was no evidence for sex interactions in most of our analyses (S7 Table); therefore, the results of the sex-stratified analyses are only mentioned when there was evidence for a sex interaction (*p* < 0.05 on likelihood ratio tests for interaction). We describe the results from analysis of multiply imputed data as our main results; results from complete case analysis were generally closer to the null than in imputed data, but the overall picture of results was similar (S7 Table).

### Association between ACEs and educational attainment

**Fig 1:**
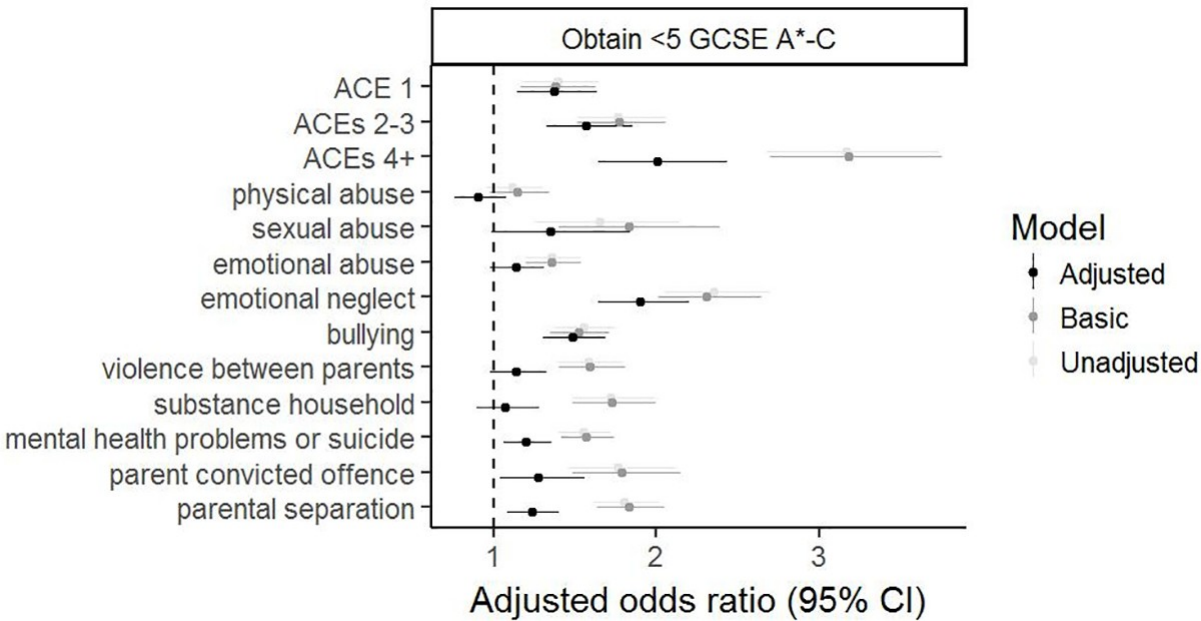
Forest plot for the associations of the ACE score and separate ACEs with obtaining fewer than 5 GCSEs at A*–C, including Maths and English. The reference category for each category of the ACE score (1, 2–3, and 4+) is experiencing 0 ACEs. The basic model is adjusted for sex. The adjusted model additionally includes home ownership, maternal and partner education, household social class, parity, ethnicity, maternal age, maternal marital status, and maternal and partner depression during pregnancy. GCSEs are qualifications obtained at age 16, which was the end of compulsory education for this cohort. ACE, adverse childhood experience; CI, confidence interval; GCSE, General Certificate of Secondary Education.

Fig 1 and S7 Table show the results for the associations of the ACE score and each individual ACE with educational attainment (3 models: unadjusted, adjusted for sex, and adjusted for confounders). Higher ACE score was associated with lower educational attainment. Associations were apparent even when comparing those experiencing only one ACE to those with no ACEs (for example, odds ratio [OR] for fewer than 5 GCSEs at grade C or above: 1.37, 95% CI: 1.15–1.63, *p* > 0.001 after adjustment for confounders) and were stronger for each increasing value of the ACE score. Experiencing 4 or more ACEs was associated with double the odds of obtaining fewer than 5 GCSEs at grade C or above (OR 2.00 [95% CI 1.65–2.43, *p* < 0.001]) after adjustment for confounders (S7 Table).

Most of the individual ACEs were associated with lower educational attainment. The strongest associations were seen for emotional neglect, for example, OR for fewer than 5 GCSEs at grade C or above 1.90 (95% CI 1.64–2.20, *p* < 0.001). Associations were weak or absent for physical abuse. For all ACEs and all measures of educational attainment, associations were considerably weaker after adjustment for confounders. For bullying, there was evidence of a sex interaction, with the associations with fewer than 5 GCSEs at grade C or above being stronger in females (1.78, 95% CI 1.48–2.15, *p* < 0.001) compared with males (1.25, 95% CI 1.05–1.50, *p* = 0.01); *p*-value for interaction 0.01 (S7 Table). Including maternal age as a categorical variable (less than 20 years, 20–34 years, or over 35 years) instead of a continuous variable, as requested by a reviewer, did not materially change the pattern of results, with ORs changing between −2.8% and 5.7%; full details are in S8 Table.

There was no consistent evidence of an interaction between ACEs and parental social class or maternal education (S9 Table and S10 Table; S1 Fig and S2 Fig) in their relationship with educational outcomes.

### Association between ACEs and health/health-related behaviours

**Fig 2:**
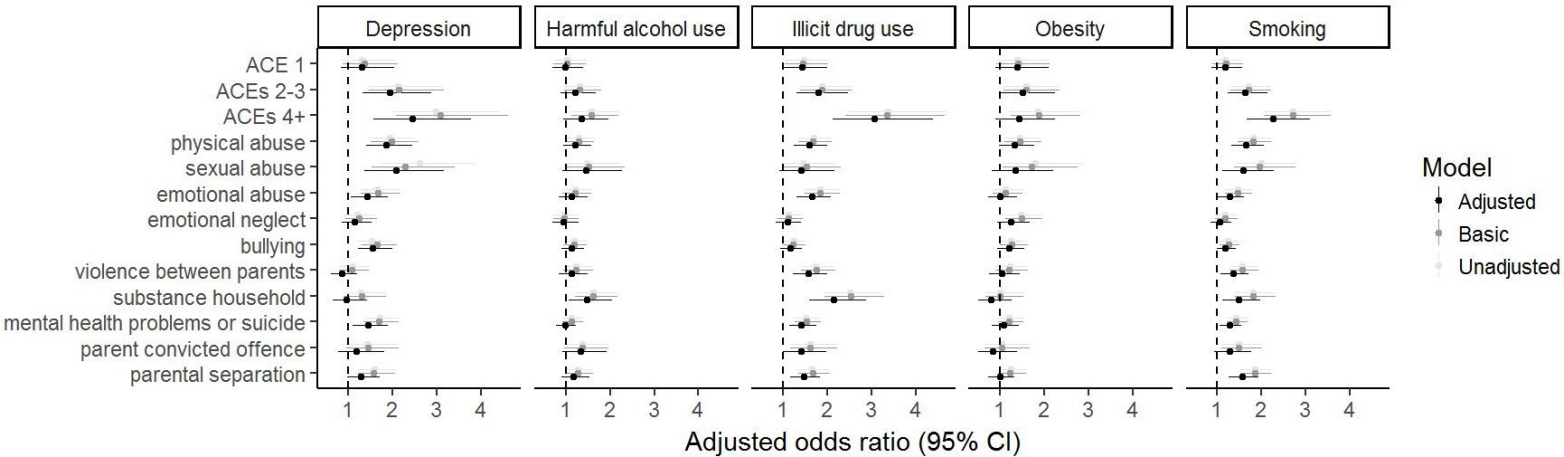
Forest plots for the associations of the ACE score and separate ACEs with poor health outcomes. The reference category for each category of the ACE score (1, 2–3, and 4+) is experiencing 0 ACEs. The basic model is adjusted for sex. The adjusted model additionally includes home ownership, maternal and partner education, household social class, parity, ethnicity, maternal age, maternal marital status, and maternal and partner depression during pregnancy. ACE, adverse childhood experience; CI, confidence interval.

The ACE score was associated with depression, illicit drug use, and smoking (Fig 2, S7 Table). The group of participants who experienced 1 ACE had higher odds of all of these outcomes compared to those who experienced no ACE, but the CIs included the null apart from for illicit drug use (OR after adjustment for confounders 1.4, 95% CI 1.0–2.0, *p* = 0.03). People who experienced 2–3 or 4 or more ACEs were more likely to be depressed, use illicit drugs, and smoke; for example, ORs for 4+ ACEs compared with no ACEs after adjustment for confounders were low educational attainment 2.0 (1.7–2.4, *p* < 0.001), depression 2.4 (1.6–3.8, *p* < 0.001), drug use 3.1 (2.1–4.4, *p* < 0.001), and smoking 2.3 (1.7–3.1, *p* < 0.001) (S7 Table).

There was only weak evidence of associations of the ACE score with obesity and harmful alcohol consumption; the OR for 4+ ACEs compared with no ACEs was 1.4 for obesity (95% CI 0.9–2.2, *p* = 0.13) and 1.4 for harmful alcohol consumption (95% CI 0.9–2.0, *p* = 0.10).

Examining individual ACEs revealed associations of i) physical abuse with depression, illicit drug use, and smoking; ii) sexual abuse with depression and smoking; iii) emotional abuse with illicit drug use and smoking; iv) bullying with depression; v) violence between parents with illicit drug use and smoking; vi) parental substance abuse with harmful alcohol use, illicit drug use, and smoking; vii) parental mental health problems with depression, illicit drug use, and smoking; and vii) parental separation with illicit drug use and smoking. Thus, overall, the patterns mirrored those of the ACE score—associations were observed for depression, illicit drug use, and smoking and were weak or absent for obesity and harmful alcohol use. The strongest associations tended to be seen for physical and sexual abuse, although very strong associations were seen for parental substance use in relation to substance use outcomes in the offspring.

In contrast with the educational outcomes, adjustment for sociodemographic confounders did not markedly attenuate the associations between ACEs and health/behavioural outcomes, and in some instances, adjustment resulted in stronger associations.

The only sex interaction was for exposure to parental substance abuse and illicit drug use (S7 Table), with stratified analyses indicating a stronger association in females than males (females, OR = 2.7, 95% CI 1.8–4.0; males, OR = 1.7, 95% CI 1.1–2.6; *p*-value for interaction 0.04).

Consistent with the educational outcomes, there was no evidence that associations between ACEs and health and behavioural outcomes differed according to parental social class or maternal education (S9 Table, S10 Table, S1 Fig and S2 Fig). Also consistent with the educational outcomes, including maternal age as a categorical variable (less than 20 years, 20–34 years, or over 35 years) instead of a continuous variable, as requested by a reviewer, did not materially change the pattern of results, with ORs changing between −4.3% and 8.5%; full details are in S8 Table.

### PAFs

Differences in the risk of achieving fewer than 5 GCSEs at grade C or above including English and Maths (Table 2) ranged from 11% (95% CI 9%–14%) for maternal depression during pregnancy to 35% (95% CI 32%–37%) for social housing. All but one sociodemographic factor (maternal depression) had RDs that were higher than for 4+ ACEs (RD 18%, 95% CI 15%–20%). The lowest PAF was for maternal age less than 20 years (2%), reflecting the low prevalence of this risk factor. The highest PAF was for maternal education (19%). This compares to 9% for 4+ ACEs.

**Table 2:** Associations of ACEs and various sociodemographic markers with educational attainment on the RD scale and PAFs. PAF is the proportion of the people experiencing <5 GCSEs (which are qualifications obtained at age 16, which was the end of compulsory education for this cohort) at grade C or above (including English and Maths) who also experienced the ‘exposure’ (ACE or sociodemographic variable). PAF can be interpreted as the proportion of the cases of <5 GCSEs at grade C or above that could be prevented if the exposure was eliminated, assuming causality. Note that the reference categories in this table differ from those in other parts of the manuscript; here, the reference category is all other participants apart from those with the exposure (for example, the reference category for 4+ ACEs here is <4 ACEs).

	*Prevalence of Exposure*	*Fewer than 5 GCSEs at Grade C or Above*, *Including English and Maths*			
		Prevalence in exposed	Prevalence in unexposed	RD (95% CI)	PAF
4 or more ACEs	19.0%	59.1%	41.2%	18% (15% to 20%)	9%
Low maternal education (CSE, vocational, or lower)	29.6%	65.6%	36.8%	29% (27% to 31%)	19%
Manual social class (III manual and lower)	22.9%	64.8%	39.4%	25% (23% to 28%)	13%
Maternal depression in pregnancy	20.5%	54.3%	43.1%	11% (9% to 14%)	5%
Any maternal smoking in pregnancy	25.7%	59.6%	40.4%	19% (17% to 21%)	11%
Social housing	14.5%	75.3%	40.4%	35% (32% to 37%)	11%
Mother aged 19 years or lower at birth	3.6%	74.6%	44.5%	30% (25% to 35%)	2%

CSE is a subject-specific qualification awarded in both academic and vocational fields in England, Wales, and Northern Ireland. CSE examinations were set in the years 1965 to 1987 inclusive. **Abbreviations:** ACE, adverse childhood experience; CI, confidence interval; CSE, Certificate of Secondary Education; GCSE, General Certificate of Secondary Education; PAF, population attributable fraction; RD, risk difference.

The highest obesity PAFs were seen for socioeconomic markers; for example, the PAFs for social housing and low maternal education were 14% and 13%, respectively, compared with 5% for 4+ ACEs (Table 3). In contrast, PAFs for illicit drug use and depression were highest for 4+ ACEs, with high PAFs also seen for maternal smoking in pregnancy and relatively low PAFs for socioeconomic and other family measures. For example, the PAFs of depression for 4+ ACEs, maternal smoking during pregnancy, and manual social class were 14%, 10%, and 3%, respectively.

**Table 3:** Associations of ACEs and various sociodemographic markers with health and health risk behaviours on the RD scale and PAFs. PAF is the proportion of the people experiencing each outcome (depression, illicit drug use, obesity, harmful alcohol use, or smoking) who also experienced the ‘exposure’ (ACEs or sociodemographic variable). PAF can be interpreted as the proportion of the outcome cases that could be prevented if the exposure was eliminated, assuming causality. Note that the reference categories in this table differ from those in other parts of the manuscript; here, the reference category is all other participants apart from those with the exposure (for example, the reference category for 4+ ACEs here is <4 ACEs).

	*Prevalence of Exposure*	Outcome Prevalence in Exposed	Outcome Prevalence in Unexposed	RD (95% CI)	PAF
***Depression***					
4 or more ACEs	16.6%	13.3%	7.5%	6% (4% to 8%)	14%
Low maternal education (CSE, vocational, or lower)	19.4%	9.0%	8.6%	0% (−2% to 2%)	1%
Manual social class (III manual and lower)	16.8%	10.0%	8.4%	2% (−1% to 4%)	3%
Maternal depression in pregnancy	17.3%	11.9%	7.9%	4% (2% to 6%)	8%
Any maternal smoking in pregnancy	17.9%	12.7%	7.8%	5% (3% to 7%)	10%
Social housing	8.5%	14.1%	8.1%	6% (3% to 9%)	6%
Mother aged 19 years or lower at birth	1.7%	19.1%	8.5%	11% (2% to 19%)	2%
***Illicit drug use***					
4 or more ACEs	16.6%	25.4%	13.7%	12% (9% to 15%)	15%
Low maternal education (CSE, vocational, or lower)	19.4%	14.6%	16.4%	−2% (−4% to 1%)	2%
Manual social class (III manual and lower)	16.8%	19.8%	15.2%	5% (2% to 7%)	5%
Maternal depression in pregnancy	17.3%	20.4%	15.1%	5% (2% to 8%)	6%
Any maternal smoking in pregnancy	17.9%	23.3%	14.4%	9% (6% to 12%)	10%
Social housing	8.5%	20.7%	15.6%	5% (1% to 9%)	3%
Mother aged 19 years or lower at birth	1.7%	26.3%	15.9%	10% (1% to 20%)	1%
***Obesity***					
4 or more ACEs	16.6%	8.7%	6.9%	2% (−0% to 4%)	5%
Low maternal education (CSE, vocational, or lower)	19.4%	11.1%	6.3%	5% (3% to 7%)	13%
Manual social class (III manual and lower)	16.8%	10.4%	6.6%	4% (2% to 6%)	9%
Maternal depression in pregnancy	17.3%	8.5%	7.0%	2% (−0% to 4%)	4%
Any maternal smoking in pregnancy	17.9%	10.5%	6.5%	4% (2% to 6%)	10%
Social housing	8.5%	17.3%	6.3%	11% (7% to 15%)	14%
Mother aged 19 years or lower at birth	1.7%	12.5%	7.2%	5% (−2% to 12%)	1%
***Harmful alcohol use***					
4 or more ACEs	16.6%	13.6%	10.3%	3% (1% to 6%)	6%
Low maternal education (CSE, vocational, or lower)	19.4%	11.5%	10.8%	1% (−2% to 3%)	1%
Manual social class (III manual and lower)	16.8%	11.9%	10.7%	1% (−1% to 4%)	2%
Maternal depression in pregnancy	17.3%	14.7%	10.1%	5% (2% to 7%)	8%
Any maternal smoking in pregnancy	17.9%	16.2%	9.8%	6% (4% to 9%)	11%
Social housing	8.5%	12.4%	10.8%	2% (−2% to 5%)	1%
Mother aged 19 years or lower at birth	1.7%	12.7%	10.9%	2% (−5% to 9%)	<1%
***Smoking***					
4 or more ACEs	16.6%	29.0%	17.1%	12% (9% to 15%)	12%
Low maternal education (CSE, vocational, or lower)	19.4%	25.1%	18.1%	7% (4% to 10%)	7%
Manual social class (III manual and lower)	16.8%	28.6%	17.5%	11% (8% to 14%)	10%
Maternal depression in pregnancy	17.3%	24.2%	18.5%	6% (3% to 9%)	5%
Any maternal smoking in pregnancy	17.9%	32.9%	16.5%	16% (13% to 20%)	15%
Social housing	8.5%	31.8%	18.3%	14% (9% to 18%)	6%
Mother aged 19 years or lower at birth	1.7%	25.1%	19.4%	6% (−4% to 15%)	1%

**Abbreviations:** ACE, adverse childhood experience; CI, confidence interval; CSE, Certificate of Secondary Education; PAF, population attributable fraction; RD, risk difference.

Harmful alcohol use and smoking exhibited a different pattern again; the highest PAF for each of these outcomes was for maternal smoking during pregnancy: 11% for harmful alcohol use and 15% for smoking. PAFs for 4+ ACEs were 6% for harmful alcohol use and 12% for smoking. Socioeconomic markers had lower PAFs for harmful alcohol use, but PAFs for smoking were similar to the PAF for 4+ ACEs, i.e., PAF for manual social class in relation to smoking was 10%.

## Discussion

In this UK cohort, in which 84% of participants were exposed to at least one ACE and 24% were exposed to 4 or more ACEs, we find evidence that ACEs—both when considered together as an ACE score and separately as individual ACEs—are associated with lower educational attainment and worse health and health-related behaviours. Adjustment for a wide range of family and socioeconomic variables reduced the magnitude of associations of ACEs with educational attainment by approximately half. However, adjusted associations between ACEs and educational attainment were still present and similar in magnitude to the strongest associations between ACEs and health/health-related behaviours. Adjustment for confounders did not attenuate associations between ACEs and health and health-related behaviours. We found no evidence that higher socioeconomic position acted as a buffer to the adverse effects of ACEs; associations between ACEs and both educational and health outcomes were similar in adolescents with parents from manual and nonmanual occupational social classes and for adolescents with low and high levels of maternal education. When calculating the proportion of cases of each outcome attributable to 4+ ACEs or various family and socioeconomic measures, we found a different pattern of results across the outcomes. For education and obesity, the highest PAFs were observed for socioeconomic markers. In contrast, PAFs for illicit drug use and depression were highest for 4+ ACEs, and for harmful alcohol use and smoking, the highest PAF was for maternal smoking during pregnancy.

Estimates of the effects of ACEs on education attenuated by half when adjusting for family and socioeconomic factors. This suggests that the family and socioeconomic context leads to considerable confounding of the association between ACEs and education. However, most studies of the sequalae of ACEs do not include the broad range of confounders that we included. Consequently, they may be overestimating the impact of ACEs. Although we adjusted for a wide array of factors, we are unlikely to have captured all relevant concepts, and our measurements will not perfectly capture the concepts of interest. For example, current housing tenure may not completely capture life course trajectories of housing tenure and does not fully capture crowding, damp, residential instability, and other important aspects of housing. Therefore, residual confounding is likely to be present, and our estimates of the educational impact of ACEs are likely to be overestimates. In contrast, our results indicate that, at least in this population, the associations of ACEs with health and health-related behaviours in adolescence were not strongly affected by adjustment for sociodemographic confounders. This suggests socioeconomic and family-level factors do not confound ACE–health associations and that ACEs are associated with these outcomes regardless of the family and sociodemographic setting in which they are experienced. The degree to which previous studies have adjusted for potential confounding variables varies considerably, with some studies making no attempt at adjustment [6]. Studies that do adjust for a range of factors differ in whether this adjustment leaves associations largely unchanged [32] or results in considerable attenuation [1].

Our a priori hypothesis was that associations between ACEs and adverse educational and health outcomes would be weaker in adolescents from high-socioeconomic–position families because other aspects of a high-socioeconomic–position environment could act to mitigate the effects of ACEs. We found no evidence to support this hypothesis; associations were of similar magnitude in manual and nonmanual social class families and in families with high and low levels of maternal education. Other studies have also found no differences in associations between ACEs and outcomes according to socioeconomic position [33] or race [32], and one study found either no difference in ACE–outcome associations according to income or stronger associations in high-income groups [15]. Together, these findings support universal ACE prevention or support interventions rather than focusing ACE initiatives only in low socioeconomic population groups.

PAFs estimate the proportion of an outcome that could be eliminated if the exposure is removed from the population, or alternatively, the proportion of people experiencing an outcome who would be reached by an intervention targeted at a particular exposure. The former interpretation of a PAF requires unrealistically strong assumptions about causality and lack of bias, but it is the latter interpretation that is of greater relevance for this study. Since the exposures we examine are interrelated, the PAFs should also not be considered in isolation because a joint PAF for all exposures considered together is likely to be considerably smaller than implied by the individual PAFs. Nonetheless, the comparative magnitude of PAFs across exposures may be informative. Our results imply that ACE-focused interventions may have less impact on population-level educational attainment compared with interventions or policies that address socioeconomic disadvantage, whereas ACE-focused initiatives may yield the greatest population-level effects on depression and drug use in adolescents. The findings for smoking and harmful alcohol use may reflect family-level propensity for risky behaviour and intergenerational transmission of behaviours. For all outcomes, PAFs were relatively low, ranging from 1% to 15% across all exposures and 5% to 15% for 4 or more ACEs. Thus, the maximum risk reduction in occurrence of the outcomes we have studied is 1%–15% for an intervention targeted at a single one of the social exposures we have considered, meaning that interventions targeting subgroups based solely on exposure to ACEs or solely on exposure to any of the socioeconomic or family-level factors we consider will fail to prevent most cases. These percentages, however, do not reflect the interrelationships between outcomes. In reality, an intervention that improves educational attainment is likely to also favourably affect health and health-related behaviours. Our findings suggest that both for research and for interventions, we should consider the broad social context and sets of exposures that contribute to adolescent health and educational outcomes.

Within the ALSPAC cohort, there is a vast amount of information, mainly prospectively collected from both young people and their mothers, from various time points and life stages. This vast array of data, considered together, resulted in a higher prevalence of many ACEs than is seen in some other studies. For example, in the Welsh ACE study, one in seven participants reported 4 or more ACEs [34], compared with one in four in ALSPAC. Studies using a single retrospective questionnaire may be underestimating the prevalence of ACEs. However, it is also possible that our cohort is identifying a set of people for whom experience of ACEs has been less severe or of shorter duration than those identified in cross-sectional studies collecting retrospective reports of ACE exposure at a single time point in adults. There is evidence that retrospective and prospective reports of ACEs capture largely nonoverlapping groups of individuals but that both groups are at risk from adverse outcomes [35, 36].

Although using data from multiple questionnaires across a long period of time enabled us to capture a detailed picture of the cohort members’ experience of ACEs, data missingness became a challenge. We assumed that the data are missing at random, given the variables included in the imputation model. Although this assumption is untestable, it allows for maximum use of the available data, and we included a number of key sociodemographic variables in the imputation model to make this assumption more plausible. The prevalence of ACEs was higher in imputed data compared with the complete case data, reflecting the fact that loss to follow-up was greater in those who have experienced ACEs. In addition to missing data for ACE measures, loss to follow-up meant that the sample size available for health outcomes (measured at a clinic at age 17 years) was much smaller than that available for education (assessed through linkage to routine data). The participants included in analysis of health outcomes are, on average, of higher socioeconomic position than those in the education analysis. In general, we would anticipate that loss to follow-up would potentially bias associations of ACEs with adverse outcomes towards the null [31]. ALSPAC is a geographical cohort based in the southwest of England and has slightly higher levels of socioeconomic advantage and lower levels of ethnic diversity than the national average, and this may affect the generalisability of our findings.

We used two ways of conceptualising ACEs: first, as a score of the number of ACEs experienced, and second, considering each ACE separately. Although the ACE score is widely used and has advantages, including the recognition that ACEs tend to co-occur and that the worst outcomes tend to be for people exposed to multiple ACEs, it is also problematic in several ways. For example, it assumes that each ACE has the same magnitude and direction of association with the outcome [37]; our analysis of individual ACEs demonstrates this not to be the case. Nonetheless, we opted to use the ACE score approach rather than alternative grouping methods such as factor analysis or latent class models [38] to be consistent with other studies. When analysing each individual ACE, we did not adjust for other ACEs as covariates. The rationale for this is that the causal structure linking multiple ACEs is complex and largely unknown. Some adjustments would therefore be overadjustment, removing some of the effect of interest.

Our results suggest associations of ACEs with lower educational attainment and worse health and health-related behaviours in late adolescence that are robust to adjustment for a wide range of variables describing the family and socioeconomic context. However, our data indicate that prevention of ACEs or improved support for people who experience ACEs, whilst beneficial, would not affect the vast majority of people experiencing adverse educational and health outcomes in adolescence. The loss of human potential associated with ACEs has led to urgent calls for ACE awareness and action to ensure that young people reach their developmental potential. Our results suggest that, whilst welcome, interventions targeted at ACE prevention/support should be considered alongside other risk factors, including socioeconomic deprivation, parental substance use, and mental health.

## Supporting information

S1 Checklist STROBE checklist for the reporting of cohort analysis. STROBE, STrengthening the Reporting of OBservational studies in Epidemiology.
(DOCX)

S1 Text ACE definitions. ACE, adverse childhood experience.
(DOCX)

S2 Text Project plan submitted to the ALSPAC for approval prior to commencement of analysis. ALSPAC, Avon Longitudinal Study of Parents and Children.
(DOCX)

S1 Fig Associations between ACEs and educational attainment (fewer than 5 GCSEs), health, or health-related behaviours (depression, harmful alcohol use, illicit drug use, obesity, smoking), stratified by parental social class. ACE, adverse childhood experience; GCSE, General Certificate of Secondary Education.
(DOCX)

S2 Fig Associations between ACEs and educational attainment (fewer than 5 GCSEs), health, or health-related behaviours (depression, harmful alcohol use, illicit drug use, obesity, smoking), stratified by maternal education. ACE, adverse childhood experience; GCSE, General Certificate of Secondary Education.
(DOCX)

S1 Table Phrasing and cutoff criteria for the ALSPAC adversity questions used to derive the ACE constructs used in this study. ACE, adverse childhood experience; ALSPAC, Avon Longitudinal Study of Parents and Children.
(DOCX)

S2 Table List of the data files (including links) used in this study. (DOCX)

S3 Table Distributions of imputed characteristics in the imputation data sets and in observed data (i.e., without imputation). AUDIT, alcohol use disorders identification test; CVA, contextual value added; EPDS, Edinburgh Postnatal Depression Scale; FSM, free school meal; GCSE, General Certificate of Secondary Education; GNVQ, General National Vocational Qualification; IDACI, Income Deprivation Affecting Children Index; SEN, special educational needs.
(DOCX)

S4 Table Distributions of outcome and ACE variables in the imputation data sets and in observed data (i.e., without imputation) in boys and girls. ACE, adverse childhood experience; AUDIT, alcohol use disorders identification test; GCSE, General Certificate of Secondary Education.
(DOCX)

S5 Table Description of the variables in the imputation model. Most variables were part of both the educational attainment as well as the health outcome imputation model, but outcome variables and certain auxiliary variables were specific to one of the two analyses (final column). ^1^The passive imputation of the ACE score was performed according to I(emotional_abuse + physical_abuse + sexual_abuse + mental_suicide_household + parental_separation + emotional_neglect+ violence_household + bullying + substance_household+ parent_convicted). ^2^None of the passively imputed variables (ACE count score variable, obesity and harmful alcohol use) were used as a predictor of missingness for the other variables in the imputation model. ACE, adverse childhood experience; AUDIT, alcohol use disorders identification test; CVA, contextual value added; EPDS, Edinburgh Postnatal Depression Scale; FSM, free school meal; GCSE, General Certificate of Secondary Education; GNVQ, General National Vocational Qualification; IDACI, Income Deprivation Affecting Children Index; SEN, special educational needs.
(DOCX)

S6 Table Associations between health outcomes. *N* = 4,917, using multiply imputed data.
(DOCX)

S7 Table AORs, 95% CIs, and *p*-values for the association between the ACE measures and educational attainment or health. The basic model was adjusted for sex, whereas the adjusted model also includes sociodemographic indicators. The *p*-value of the interaction test is for the comparison of the original model with a model including a sex interaction. Furthermore, model estimates of sex-stratified analyses are given. ACE, adverse childhood experience; AOR, adjusted odds ratio; CI, confidence interval.
(DOCX)

S8 Table AORs, 95% CIs, and *p*-values for the association between the ACE measures and educational attainment or health for the sensitivity analysis with maternal age as a categorical variable (less than 20 years, 20–34 years, or over 35 years). The basic model was adjusted for sex, whereas the adjusted model also includes sociodemographic indicators. For convenience, the AOR for the models with a continuous maternal age variable (AORcon) as well as percentage of difference in the AOR are also included. ACE, adverse childhood experience; AOR, adjusted odds ratio; CI, confidence interval.
(DOCX)

S9 Table Estimated regression coefficients (for continuous outcomes) or AORs (for categorical outcomes), 95% CIs, and *p*-values for the interaction between each ACE and parental social class. ACE, adverse childhood experience; AOR, adjusted odds ratio; CI, confidence interval.
(DOCX)

S10 Table Estimated regression coefficients (for continuous outcomes) or AORs (for categorical outcomes), 95% CIs, and *p*-values for the interaction between each ACE and maternal education. ACE, adverse childhood experience; AOR, adjusted odds ratio; CI, confidence interval.
(DOCX)

## Abbreviations

ACE: adverse childhood experience
ALSPAC: Avon Longitudinal Study of Parents and Children
AUDIT: alcohol use disorders identification test
BMI: body mass index
CI: confidence interval
CIS-R: clinical interview schedule—revised
EPDS: Edinburgh Postnatal Depression Scale
E-Risk: Environmental Risk
GCSE: General Certificate of Secondary Education
NPD: National Pupil Database
OR: odds ratio
RD: risk difference
STROBE: STrengthening the Reporting of OBservational studies in Epidemiology
