# Supplementary material for: Associations of adverse childhood experiences with educational attainment and adolescent health and the role of family and socioeconomic factors: A prospective cohort study in the UK
Source: PLoS Med. 2020 Mar 2;17(3):e1003031. doi: 10.1371/journal.pmed.1003031 (PMC7051040; doi:10.1371/journal.pmed.1003031)
Supplement: S1 Text — ACE, adverse childhood experience. (DOCX) [file pmed.1003031.s002.docx]

***S1 Text Adverse childhood experiences (ACE) definitions***

The cut-offs for each individual question are described in (1). In short, the adverse childhood experiences were defined as:

1. adult in family was ever physically cruel towards or hurt the child (*physical abuse*);
2. ever sexually abused, forced to perform sexual acts or touch someone in a sexual way (*sexual abuse*);
3. parent was ever emotional cruel towards child or often said hurtful/insulting things to the child (*emotional abuse*);
4. child always felt excluded, misunderstood or never important to family, parents never asked or never listened when child talked about their free time (*emotional neglect*);
5. child was a victim of bullying on a weekly basis (*bullying*);
6. parents were ever affected by physically cruel behaviour by partner, or, ever violent towards each other, including hitting, choking, strangling, beating, shoving (*violence between parents*);
7. parent was a daily cannabis or any hard drug user, or, had an alcohol problem (*substance use in household*);
8. parent was ever diagnosed with schizophrenia or hospitalised for a psychiatric problem, or, during the first 16 years of the child’s life, parent had an eating disorder (bulimia or anorexia), used medication for depression or anxiety, attempted to commit suicide or scored above previously established cut-offs for depression (Edinburgh Postnatal Depression Scale (EPDS) >12 (2)) (*mental health problems or suicide*);
9. parent was convicted of an offence (*parent convicted*);
10. parents separated or divorced (*parental separation*);

**References**

1.  Houtepen LC, Heron J, Suderman MJ, Tilling K, Howe LD. Adverse childhood experiences in the children of the Avon Longitudinal Study of Parents and Children (ALSPAC). Wellcome Open Res. 2018 Aug 30;3:106. doi: 10.12688/wellcomeopenres.14716.1. eCollection 2018;

2.  Pearson RM, Evans J, Kounali D, Lewis G, Heron J, Ramchandani PG, et al. Maternal depression during pregnancy and the postnatal period: risks and possible mechanisms for offspring depression at age 18 years. JAMA psychiatry [Internet]. 2013;70(12):1312–9. Available from: http://www.ncbi.nlm.nih.gov/pubmed/24108418
