# Supplementary material for: Associations of adverse childhood experiences with educational attainment and adolescent health and the role of family and socioeconomic factors: A prospective cohort study in the UK
Source: PLoS Med. 2020 Mar 2;17(3):e1003031. doi: 10.1371/journal.pmed.1003031 (PMC7051040; doi:10.1371/journal.pmed.1003031)
Supplement: S2 Text — ALSPAC, Avon Longitudinal Study of Parents and Children. (DOCX) [file pmed.1003031.s003.docx]

**Project plan submitted to the Avon Longitudinal Study of Parents and Children for approval prior to commencement of analysis**

Title of project: Early life adversity and educational attainment

Project summary for laypersons:

Early life adversity (e.g. abuse, neglect, parental illness, parental death, parental separation) is associated with a range of poor outcomes in later life, including both social and health outcomes. This project will investigate the association between early life adversity and educational attainment.

Aim(s) and objective(s):

Aim: To investigate the association between early life adversity and educational attainment
Objectives:
1. To assess whether the association differs according to type of adversity experienced
2. To assess whether the association differs according to the age at which adversity was experienced
3. To assess whether the association differs in males and females and across socioeconomic groups
4. To assess whether experiencing multiple forms of early life adversity is particularly detrimental for educational attainment

Methods (including an overview of statistical methods):

We will use linear regression to assess associations of interest, adjusting for potential confounders, and using interaction tests to assess whether associations differ according to gender and SEP. We will assess the associations for each separate form of early life adversity, and for a summary score of the number of types of adversity experienced.

Exposures, outcomes and confounders to be considered (justifying particular types of data as necessary)

Exposures - early life adversity measures: emotional neglect, sexual abuse, physical abuse, emotional abuse, violence in the household, substance abuse by parents, parental mental illness, parental loss, bullying, maternal-child bonding, neighbourhood-level safety, social support, intimate partner violence, parents being in trouble with the law. These measures are reported in questionnaires by the mothers and the children

Outcomes - educational attainment: key stage exam results from record linkage (all available), self-reported continuation into higher education from child completed questionnaires

Confounders - maternal and paternal education and social class, housing tenure, financial difficulties as reported in mother-completed questionnaires
