# Supplementary material for: Associations of adverse childhood experiences with educational attainment and adolescent health and the role of family and socioeconomic factors: A prospective cohort study in the UK
Source: PLoS Med. 2020 Mar 2;17(3):e1003031. doi: 10.1371/journal.pmed.1003031 (PMC7051040; doi:10.1371/journal.pmed.1003031)
Supplement: S1 Fig — ACE, adverse childhood experience; GCSE, General Certificate of Secondary Education. (DOCX) [file pmed.1003031.s004.docx]

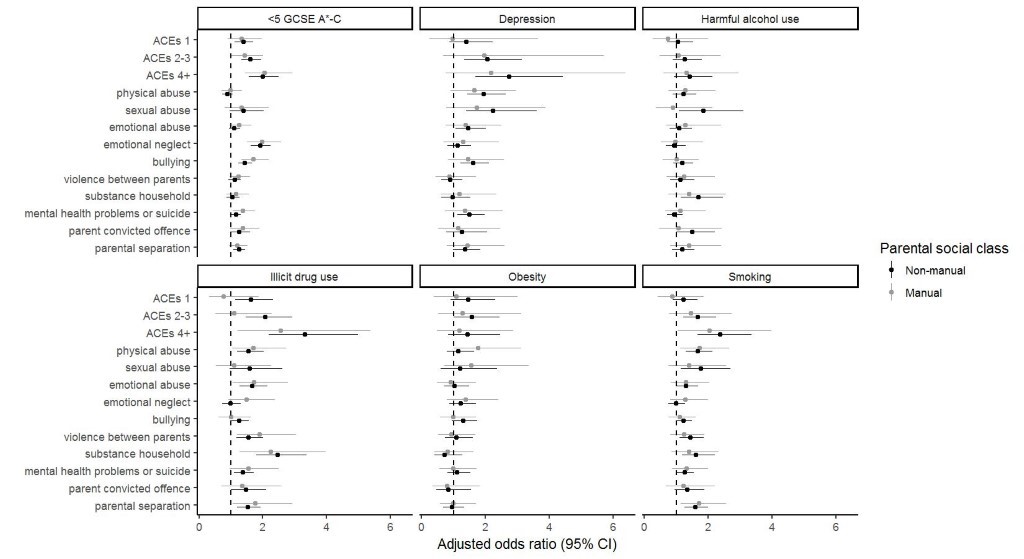


S1 Fig: Associations between ACE and educational attainment (less than 5 GCSEs), health or health-related behaviours (Depression, Harmful alcohol use, Illicit drug use, Obesity, Smoking), stratified by parental social class.
