# Supplementary material for: Associations of adverse childhood experiences with educational attainment and adolescent health and the role of family and socioeconomic factors: A prospective cohort study in the UK
Source: PLoS Med. 2020 Mar 2;17(3):e1003031. doi: 10.1371/journal.pmed.1003031 (PMC7051040; doi:10.1371/journal.pmed.1003031)
Supplement: S1 Table — ACE, adverse childhood experience; ALSPAC, Avon Longitudinal Study of Parents and Children. (DOCX) [file pmed.1003031.s006.docx]

*S1 Table Phrasing and cut-off criteria for the ALSPAC adversity questions used to derive the ACE constructs used in this study*

| **Adverse childhood experience (ACE)** | **Phrasing** | **Criterion** | **Retrospective** |  |  |  |  |
| --- | --- | --- | --- | --- | --- | --- | --- |
|  |  |  |  | Physical abuse | Partner/respondent  wasphysically cruel tochild | yes | no |
|  |  |  |  |  | Adult infamilypushed, grabbed, shoved/smacked to discipline respondent, beforeageof 11 | often | yes (asked at 22yrs) |
|  |  |  |  |  | When growing up people in respondent's family hit them so hard that it left them with bruises or marks | yes | yes (asked at 23yrs) |
| Adult in family kicked, punched, hit respondent (so hard it left bruises or marks), before age of 11 | yes | yes (asked at 22yrs) |  |  |  |  |  |
| Sexual abuse | Sexually abused | yes | no |  |  |  |  |
|  | When growing up someone molested respondent (sexually) | yes | yes (asked at 23yrs) |  |  |  |  |
|  | Touched in a sexual way by adult or older child, or was forced to touch adult or older child in a sexual way, before age of 11 | yes | yes (asked at 22yrs) |  |  |  |  |
|  | Adult or older child forced, or attempted to force, respondent into any sexual activity by threatening or holding respondent down or hurting respondent in some way, before age of 11 | yes | yes (asked at 22yrs) |  |  |  |  |
| Emotional abuse | Partner/respondent was emotionally cruel to child | yes | no |  |  |  |  |
|  | Adult in family shouted/ said hurtful or insulting things to respondent, before age of 11 | Very often | yes (asked at 22yrs) |  |  |  |  |
| Emotional neglect | Carer knows who friends are | never | no |  |  |  |  |
|  | Carer asks/starts conversation about free time/ what happened at school | never | no |  |  |  |  |
|  | Carer takes time to listen when teenager talks about what happened in free time | never | no |  |  |  |  |
|  | Discuss problems with anyone in their family | very difficult | no |  |  |  |  |
|  | Parent/carer talked about child's experiences at school/ friends/ things that are troubling | never | no |  |  |  |  |
|  | Child feels left out of things | always | no |  |  |  |  |
|  | Understood by parents | not | no |  |  |  |  |
|  | When growing up there was someone to take respondent to the doctor if needed | never | yes (asked at 23yrs) |  |  |  |  |
|  | Someone in family made child feel important or special, before 11 | never | yes (asked at 22yrs) |  |  |  |  |
|  | Carer knows what child does with other children | nothing | no |  |  |  |  |
| Bullying | Overt bullying victim items including: personal belongings stolen, threatened/blackmailed, hit/beaten up | weekly | no |  |  |  |  |
|  | Relational bullying victim items including: do something didn't want to, told lies about child | weekly | no |  |  |  |  |
|  | Friends tried to get teenager to do things didn't want to / told lies about teenager | weekly | no |  |  |  |  |
|  | Child has been bullied | all the time | no |  |  |  |  |
|  | Upset by name calling/exclusion from groups or bullying | Most days | no |  |  |  |  |
|  | Someone threatened/blackmailed teenager | weekly | no |  |  |  |  |
| Violence between parents | Physically cruel | yes, affected them | yes, the reported age can be retrospective |  |  |  |  |
|  | Kicked, bitten or hit each other | yes | no |  |  |  |  |
|  | Physically twisted arm | yes | no |  |  |  |  |
|  | Throw(n) bodily | yes | no |  |  |  |  |
|  | Beaten each other up | yes | no |  |  |  |  |
|  | Choke or strangle each other | yes | no |  |  |  |  |
|  | Threatened each other with knife | yes | no |  |  |  |  |
|  | Used knife or other weapon on each other | yes | no |  |  |  |  |
| Substance abuse in household | Smoked cannabis | every day | no |  |  |  |  |
|  | Hard drug use (including crack, heroin, amphetamine, opiate, cocaine, methadone, meth) | yes | no |  |  |  |  |
|  | Hard drug addiction | yes, recently | no |  |  |  |  |
|  | Alcoholism/ Drink problem | yes, ever / yes, saw doctor | no |  |  |  |  |
|  | Alcohol Use Disorders Identification Test (AUDIT) score | >8 | no |  |  |  |  |
| Parental mental health  problems or attempted suicide | Parent has hurt themselves on purpose | yes | no |  |  |  |  |
|  | Parent has attempted suicide | yes | yes, the reported age can be retrospective |  |  |  |  |
|  | Taken medication for anxiety or depression | yes | no |  |  |  |  |
|  | Edinburgh Postnatal Depression Scale (EPDS) | >12 | no |  |  |  |  |
|  | Schizophrenia | yes, either current or ever | Both |  |  |  |  |
|  | Bulimia, anorexia nervosa | yes, recently | Both |  |  |  |  |
|  | Ever admitted to hospital for psychiatric or mental health problems | yes | yes |  |  |  |  |
| Parent convicted offence | Court conviction | yes | no |  |  |  |  |
|  | Convicted of an offence | yes | yes, the reported age can be retrospective |  |  |  |  |
| Parental separation | Parent reports divorce/separation | yes | no |  |  |  |  |
|  | Your parents have divorced/separated | yes | no |  |  |  |  |
|  | Parent still has the same partner/husband | no | no |  |  |  |  |
