# Supplementary material for: Associations of adverse childhood experiences with educational attainment and adolescent health and the role of family and socioeconomic factors: A prospective cohort study in the UK
Source: PLoS Med. 2020 Mar 2;17(3):e1003031. doi: 10.1371/journal.pmed.1003031 (PMC7051040; doi:10.1371/journal.pmed.1003031)
Supplement: S2 Table — (DOCX) [file pmed.1003031.s007.docx]

S2 Table: List of the questionnaires used in this study

| **Reported by whom (child based/child completed/carer/father/school)** | **Alspac file id** | **Available at** |
| --- | --- | --- |
| Carer | a | <http://www.bristol.ac.uk/media-library/sites/alspac/migrated/documents/ques-m01-your-environment.pdf> |
| Carer | b | http://www.bristol.ac.uk/media-library/sites/alspac/migrated/documents/ques-m03-having-a-baby.pdf |
| Carer | c | http://www.bristol.ac.uk/media-library/sites/alspac/migrated/documents/ques-m04-your-pregnancy.pdf |
| Carer | e | http://www.bristol.ac.uk/media-library/sites/alspac/migrated/documents/ques-m05-me-and-my-baby.pdf |
| Carer | f | http://www.bristol.ac.uk/media-library/sites/alspac/migrated/documents/ques-m06-looking-after-the-baby.pdf |
| Carer | g | http://www.bristol.ac.uk/media-library/sites/alspac/migrated/documents/ques-m07-caring-for-a-toddler.pdf |
| Carer | h | http://www.bristol.ac.uk/media-library/sites/alspac/migrated/documents/ques-m08-your-health-events-and-feelings.pdf |
| Carer | j | http://www.bristol.ac.uk/media-library/sites/alspac/migrated/documents/ques-m09-mothers-new-questionnaire.pdf |
| Carer | k | http://www.bristol.ac.uk/media-library/sites/alspac/migrated/documents/ques-m10-study-mothers-questionnaire.pdf |
| Carer | l | http://www.bristol.ac.uk/media-library/sites/alspac/migrated/documents/ques-m11-mothers-lifestyle.pdf |
| Carer | m | http://www.bristol.ac.uk/media-library/sites/alspac/migrated/documents/ques-m12-mother-and-home.pdf |
| Carer | n | http://www.bristol.ac.uk/media-library/sites/alspac/migrated/documents/ques-m13-mother-and-family.pdf |
| Carer | p | http://www.bristol.ac.uk/media-library/sites/alspac/migrated/documents/ques-m14-mother-of-a-9-year-old.pdf |
| Carer | q | http://www.bristol.ac.uk/media-library/sites/alspac/migrated/documents/ques-m15-you-and-your-surroundings.pdf |
| Carer | r | http://www.bristol.ac.uk/media-library/sites/alspac/migrated/documents/ques-m16-lifestyle-and-health-of-mother.pdf |
| Carer | s | http://www.bristol.ac.uk/media-library/sites/alspac/migrated/documents/ques-m17-twelve-years-on.pdf |
| Child Based | kb | http://www.bristol.ac.uk/media-library/sites/alspac/migrated/documents/ques-cb02-my-daughter.pdf |
| Child Based | kd | http://www.bristol.ac.uk/media-library/sites/alspac/migrated/documents/ques-cb04-girl-toddler.pdf |
| Child Based | kj | http://www.bristol.ac.uk/media-library/sites/alspac/migrated/documents/ques-cb08-my-sons-health-and-behaviour.pdf |
| Child Based | kl | http://www.bristol.ac.uk/media-library/sites/alspac/migrated/documents/ques-cb10-development-and-health-of-my-son.pdf |
| Child Based | kn | http://www.bristol.ac.uk/media-library/sites/alspac/migrated/documents/ques-cb12-my-school-boy.pdf |
| Child Based | kq | http://www.bristol.ac.uk/media-library/sites/alspac/migrated/documents/ques-cb14-my-son-at-school.pdf |
| Child Based | ks | http://www.bristol.ac.uk/media-library/sites/alspac/migrated/documents/ques-cb17-my-sons-health.pdf |
| Child Based | kt | http://www.bristol.ac.uk/media-library/sites/alspac/migrated/documents/ques-cb18-my-daughter-at-home-and-at-school.pdf |
| Child Based | ta | http://www.bristol.ac.uk/media-library/sites/alspac/migrated/documents/ques-cb25-my-teenage-son.pdf |
| Child Based | txa | http://www.bristol.ac.uk/media-library/sites/alspac/migrated/documents/ques-cb30-y11carer.pdf |
| Child Completed | ccc | http://www.bristol.ac.uk/media-library/sites/alspac/migrated/documents/ques-c08-me-and-my-school.pdf |
| Child Completed | ccf | http://www.bristol.ac.uk/media-library/sites/alspac/migrated/documents/ques-c11-my-hands-my-feet-and-me.pdf |
| Child Completed | ccr | http://www.bristol.ac.uk/media-library/sites/alspac/migrated/documents/ques-q21-life-of-a-teenager.pdf |
| Child Completed | ccs | http://www.bristol.ac.uk/media-library/sites/alspac/documents/questionnaires/CCS-life-of-a-16-plus-teenager.pdf |
| Child Completed | ccxa | http://www.bristol.ac.uk/media-library/sites/alspac/migrated/documents/ques-c26-y11yp.pdf |
| Child Completed | ypb | http://www.bristol.ac.uk/media-library/sites/alspac/documents/questionnaires/YPB-life-at-22-plus.pdf |
| Child Completed | ypc | http://www.bristol.ac.uk/media-library/sites/alspac/documents/questionnaires/YPC-me-at-23.pdf |
| Partner | pb | http://www.bristol.ac.uk/media-library/sites/alspac/migrated/documents/ques-p02-partners-questionnaire.pdf |
| Partner | pc | http://www.bristol.ac.uk/media-library/sites/alspac/migrated/documents/ques-p03-being-a-father.pdf |
| Partner | pd | http://www.bristol.ac.uk/media-library/sites/alspac/migrated/documents/ques-p04-the-baby-and-me.pdf |
| Partner | pe | http://www.bristol.ac.uk/media-library/sites/alspac/migrated/documents/ques-p05-a-toddler-in-the-house.pdf |
| Partner | pf | http://www.bristol.ac.uk/media-library/sites/alspac/migrated/documents/ques-p06-partners-health-events-and-feelings.pdf |
| Partner | pg | http://www.bristol.ac.uk/media-library/sites/alspac/migrated/documents/ques-p07-partners-new-questionnaire.pdf |
| Partner | ph | http://www.bristol.ac.uk/media-library/sites/alspac/migrated/documents/ques-p08-study-partners-questionnaire.pdf |
| Partner | pk | http://www.bristol.ac.uk/media-library/sites/alspac/migrated/documents/ques-p10-partner-and-home.pdf |
| Partner | pl | http://www.bristol.ac.uk/media-library/sites/alspac/migrated/documents/ques-p11-father-and-family.pdf |
| Partner | pm | http://www.bristol.ac.uk/media-library/sites/alspac/migrated/documents/ques-p12-father-of-a-9-year-old.pdf |
| Partner | pn | http://www.bristol.ac.uk/media-library/sites/alspac/migrated/documents/ques-p13-surroundings-of-husband-partner.pdf |
| Partner | pp | http://www.bristol.ac.uk/media-library/sites/alspac/migrated/documents/ques-p14-lifestyle-and-health-of-partner.pdf |
| Partner | pq | http://www.bristol.ac.uk/media-library/sites/alspac/migrated/documents/ques-p15-partner-about-me.pdf |
| Schools | sa | http://www.bristol.ac.uk/media-library/sites/alspac/migrated/documents/ques-s01-childs-behaviour-and-abilities.pdf |
| Schools | se | http://www.bristol.ac.uk/media-library/sites/alspac/migrated/documents/ques-s05-the-developing-child.pdf |
