# Supplementary material for: Associations of adverse childhood experiences with educational attainment and adolescent health and the role of family and socioeconomic factors: A prospective cohort study in the UK
Source: PLoS Med. 2020 Mar 2;17(3):e1003031. doi: 10.1371/journal.pmed.1003031 (PMC7051040; doi:10.1371/journal.pmed.1003031)
Supplement: S3 Table — AUDIT, alcohol use disorders identification test; CVA, contextual value added; EPDS, Edinburgh Postnatal Depression Scale; FSM, free school meal; GCSE, General Certificate of Secondary Education; GNVQ, General National Vocational Qualification; IDACI, Income Deprivation Affecting Children Index; SEN, special educational needs. (DOCX) [file pmed.1003031.s008.docx]

*S3 Table Distributions of imputed characteristics in the imputation datasets and in observed data (i.e. without imputation).*

| **Variable** | **Analysis 1: Education** | | | **Analysis 2: Health** | | |
| --- | --- | --- | --- | --- | --- | --- |
|  | **% data imputed** | **Distribution**  Mean (SE) for continuous variables   % for categorical variables   In | | **% data imputed** | **Distribution**  Mean (SE) for continuous variables   % for categorical variables   In | |
|  |  | Imputed | observed |  | imputed | observed |
| **OUTCOME** | | | | | | |
| <5 GCSEs including math and English at grades A*-C | 0.2 | 45.5 (4535/9959) | 45.5 (4535/9959) | n/a | n/a | n/a |
| BMI-Z at age 17 | n/a | n/a | n/a | 1.8 | 0.39 (0.02) | 0.39 (0.02) |
| AUDIT score at age 17 | n/a | n/a | n/a | 19.9 | 8.35 (0.08) | 8.15 (0.09) |
| Obesity at age 17 | n/a | n/a | n/a | 1.8 | 7.3 (357/4917) | 7.2 (350/4829) |
| Depression at age 17 | n/a | n/a | n/a | 10.6 | 8.7 (426/4917) | 7.9 (349/4395) |
| Smoking at age 17 | n/a | n/a | n/a | 17.4 | 19.5 (958/4917) | 16.9 (688/4061) |
| Illicit drug use at age 17 | n/a | n/a | n/a | 19.1 | 16.1 (790/4917) | 13.7 (545/3978) |
| Harmful alcohol use at age 17 | n/a | n/a | n/a | 19.9 | 10.9 (538/4917) | 10 (394/3939) |
| **ADVERSE CHILDHOOD EXPERIENCES** | | | | | | |
| Categorical ACE-score | 69.3 |  |  | 44.3 |  |  |
| 0 |  | 16.1 (1605/9959) | 22.1 (676/3056) |  | 18.2 (893/4917) | 22.4 (613/2741) |
| 1 |  | 23.6 (2350/9959) | 28.1 (858/3056) |  | 25.5 (1253/4917) | 29.1 (799/2741) |
| 2 3 |  | 36.5 (3634/9959) | 35.5 (1086/3056) |  | 36.1 (1775/4917) | 35.2 (966/2741) |
| 4+ |  | 23.8 (2370/9959) | 14.3 (436/3056) |  | 20.3 (996/4917) | 13.2 (363/2741) |
| physical abuse | 46.4 | 19 (1888/9959) | 15 (800/5339) | 27.6 | 20.8 (1021/4917) | 17.4 (618/3560) |
| sexual abuse | 23.9 | 4.1 (411/9959) | 2.8 (210/7576) | 12 | 5 (245/4917) | 3.9 (167/4325) |
| emotional abuse | 42.4 | 23.9 (2378/9959) | 19.2 (1100/5735) | 25.7 | 22.8 (1122/4917) | 19 (693/3653) |
| emotional neglect | 50.9 | 23.9 (2381/9959) | 19.3 (945/4887) | 17.2 | 19.8 (975/4917) | 18.2 (743/4073) |
| bullying | 39.5 | 26.2 (2607/9959) | 24.2 (1458/6028) | 10.9 | 26.9 (1322/4917) | 26 (1141/4382) |
| violence between parents | 46.8 | 25.3 (2525/9959) | 19.3 (1020/5294) | 28.5 | 21.6 (1064/4917) | 17.1 (602/3515) |
| substance household | 38.7 | 15.1 (1502/9959) | 9.5 (581/6100) | 24 | 12.2 (602/4917) | 8.4 (315/3739) |
| mental health problems or suicide | 38.5 | 48.6 (4840/9959) | 43.4 (2658/6120) | 21.8 | 44.8 (2202/4917) | 41.1 (1581/3843) |
| parent convicted offence | 36.5 | 10.5 (1050/9959) | 7.2 (452/6321) | 21.6 | 9.2 (452/4917) | 6.7 (260/3857) |
| parental separation | 45.2 | 33.8 (3371/9959) | 25.8 (1407/5454) | 27.8 | 28.3 (1391/4917) | 22.8 (811/3550) |
| **COVARIATES***basic model* | | | | | | |
| Sex (Male) | 0 | 49.6 (4936/9959) | 49.6 (4936/9959) | 0 | 56 (2754/4917) | 56 (2754/4917) |
| **COVARIATES***adjusted model* | | | | | | |
| Ethnicity child (non-white) | 9.9 | 5 (496/9959) | 4.1 (366/8978) | 8.3 | 5.3 (262/4917) | 4.3 (195/4511) |
| Household social class at 18wks gestation | 10.5 |  |  | 9 |  |  |
| I - Professional |  | 8.4 (837/9959) | 8.2 (729/8912) |  | 12.6 (620/4917) | 12.9 (577/4476) |
| II - Managerial and technical |  | 36.4 (3623/9959) | 37.5 (3344/8912) |  | 41.2 (2028/4917) | 42.7 (1910/4476) |
| IIINM - Skilled non-manual |  | 31.3 (3112/9959) | 32.5 (2892/8912) |  | 28.3 (1390/4917) | 28.5 (1276/4476) |
| IIIM - Skilled manual |  | 13.7 (1367/9959) | 13.3 (1183/8912) |  | 10.7 (527/4917) | 10 (448/4476) |
| IV - Partly skilled |  | 8 (793/9959) | 6.9 (619/8912) |  | 5.9 (291/4917) | 5.1 (227/4476) |
| V - Unskilled |  | 2.3 (227/9959) | 1.6 (145/8912) |  | 1.2 (60/4917) | 0.8 (38/4476) |
| Home ownership mother during pregnancy | 6.8 |  |  | 6.7 |  |  |
| Mortgaged |  | 73.3 (7302/9959) | 75.3 (6990/9284) |  | 80.7 (3968/4917) | 82.5 (3783/4588) |
| Owned |  | 2.3 (228/9959) | 2.1 (196/9284) |  | 2.3 (115/4917) | 2.2 (100/4588) |
| Council rented |  | 12.7 (1265/9959) | 12.2 (1131/9284) |  | 8 (394/4917) | 7.3 (335/4588) |
| Furnished private rental |  | 3.8 (378/9959) | 3.4 (318/9284) |  | 2.9 (144/4917) | 2.6 (121/4588) |
| Unfurnished private rental |  | 2.9 (287/9959) | 2.6 (243/9284) |  | 2 (99/4917) | 1.8 (83/4588) |
| Housing authority rented |  | 1.8 (176/9959) | 1.4 (131/9284) |  | 1 (50/4917) | 0.8 (38/4588) |
| Other |  | 3.2 (322/9959) | 3 (275/9284) |  | 3 (148/4917) | 2.8 (128/4588) |
| Marital status mother during pregnancy | 6.3 |  |  | 6 |  |  |
| Never married |  | 18.4 (1835/9959) | 17.2 (1604/9335) |  | 14.6 (719/4917) | 13.7 (634/4623) |
| Widowed |  | 0.2 (18/9959) | 0.1 (11/9335) |  | 0.1 (7/4917) | 0.1 (3/4623) |
| Divorced |  | 4.1 (410/9959) | 3.9 (360/9335) |  | 3.7 (183/4917) | 3.5 (161/4623) |
| Separated |  | 1.7 (170/9959) | 1.4 (128/9335) |  | 1.4 (70/4917) | 1.2 (57/4623) |
| 1st marriage |  | 69.2 (6891/9959) | 71.1 (6639/9335) |  | 73.9 (3635/4917) | 75.4 (3487/4623) |
| Marriage 2 or 3 |  | 6.4 (636/9959) | 6.4 (593/9335) |  | 6.2 (303/4917) | 6.1 (281/4623) |
| Self-reported highest educational level mother | 7.9 |  |  | 6.9 |  |  |
| Certificate of Secondary Education (CSE) |  | 19.7 (1965/9959) | 18.9 (1737/9176) |  | 12.2 (600/4917) | 11.4 (524/4578) |
| Vocational |  | 10.6 (1056/9959) | 10.2 (932/9176) |  | 7.8 (382/4917) | 7.4 (341/4578) |
| O level |  | 35.4 (3523/9959) | 36.7 (3369/9176) |  | 33.8 (1664/4917) | 34.3 (1570/4578) |
| A level |  | 22.2 (2215/9959) | 22.7 (2083/9176) |  | 27.4 (1346/4917) | 28 (1281/4578) |
| Degree |  | 12.1 (1200/9959) | 11.5 (1055/9176) |  | 18.8 (927/4917) | 18.8 (862/4578) |
| Mother-reported highest educational level partner | 11.3 |  |  | 9.2 |  |  |
| CSE |  | 27 (2691/9959) | 25.2 (2226/8835) |  | 19.9 (978/4917) | 17.9 (800/4464) |
| Vocational |  | 9.2 (921/9959) | 8.9 (790/8835) |  | 7.7 (377/4917) | 7.4 (330/4464) |
| O level |  | 21.9 (2183/9959) | 22.5 (1991/8835) |  | 20.9 (1027/4917) | 21.3 (951/4464) |
| A level |  | 25.3 (2518/9959) | 26.8 (2367/8835) |  | 27.6 (1357/4917) | 28.6 (1276/4464) |
| Degree |  | 16.5 (1646/9959) | 16.5 (1461/8835) |  | 24 (1178/4917) | 24.8 (1107/4464) |
| Maternal age in years at delivery | 3.3 | 28.23 (0.05) | 28.26 (0.05) | 4.3 | 29.23 (0.07) | 29.27 (0.07) |
| Parity | 7.2 | 0.83 (0.01) | 0.83 (0.01) | 7.1 | 0.75 (0.01) | 0.74 (0.01) |
| Maternal depression score (EPDS) at 18 wks gestation | 13.3 | 6.99 (0.05) | 6.79 (0.05) | 12.5 | 6.49 (0.07) | 6.36 (0.07) |
| Maternal depression score (EPDS) at 32 wks gestation | 10.8 | 7.11 (0.05) | 6.91 (0.05) | 9.6 | 6.66 (0.07) | 6.53 (0.07) |
| Partner depression score (EPDS) at 18 wks gestation | 28 | 4.36 (0.04) | 4.12 (0.05) | 24 | 4.25 (0.06) | 4.03 (0.06) |
| **IMPUTATION VARIABLES** | | | | | | |
| social class | 52.5 | 13.3 (1326/9959) | 10.1 (476/4733) | 52.2 | 13.5 (662/4917) | 7.7 (181/2351) |
| financial difficulties | 36.7 | 19.6 (1950/9959) | 13.8 (868/6301) | 22.3 | 15.3 (753/4917) | 11.1 (426/3822) |
| satisfaction with neighbourhood | 26.3 | 11.5 (1150/9959) | 9.2 (677/7342) | 7.8 | 11.4 (561/4917) | 10.4 (470/4535) |
| social support of child | 42.5 | 13.7 (1363/9959) | 10.5 (602/5731) | 10.8 | 11.3 (554/4917) | 10.4 (458/4388) |
| social support of parent | 34 | 14.6 (1458/9959) | 11.1 (728/6568) | 20.3 | 13 (641/4917) | 10.4 (407/3921) |
| violence between child and partner | 65 | 16.7 (1660/9959) | 11 (383/3481) | 46.7 | 13.4 (657/4917) | 10 (263/2622) |
| physical illness of the child | 21.9 | 10 (999/9959) | 8.7 (677/7780) | 12.3 | 8.9 (440/4917) | 8 (343/4311) |
| physical illness of a parent | 51.2 | 28 (2790/9959) | 24 (1169/4861) | 34.1 | 27 (1328/4917) | 24.3 (788/3240) |
| parent-child bond | 43.2 | 23.7 (2359/9959) | 19.3 (1094/5660) | 24.9 | 22.7 (1115/4917) | 19.5 (719/3695) |
| Self-reported highest educational level partner | 27.7 |  |  | 23.5 |  |  |
| CSE |  | 23.9 (2382/9959) | 20.8 (1502/7204) |  | 17.9 (880/4917) | 14.7 (551/3761) |
| Vocational |  | 10.2 (1018/9959) | 9 (651/7204) |  | 8.1 (398/4917) | 6.7 (253/3761) |
| O level |  | 22.4 (2231/9959) | 23.7 (1704/7204) |  | 22 (1082/4917) | 22.2 (834/3761) |
| A level |  | 26.2 (2608/9959) | 28.6 (2063/7204) |  | 27.6 (1359/4917) | 29.6 (1114/3761) |
| Degree |  | 17.3 (1719/9959) | 17.8 (1284/7204) |  | 24.4 (1199/4917) | 26.8 (1009/3761) |
| Partner-reported highest educational level mother | 29.4 |  |  | 25 |  |  |
| CSE |  | 22.2 (2207/9959) | 19.6 (1376/7034) |  | 14.9 (734/4917) | 11.8 (437/3688) |
| Vocational |  | 10.5 (1048/9959) | 9.8 (692/7034) |  | 8.3 (408/4917) | 7.2 (266/3688) |
| O level |  | 32.7 (3255/9959) | 34.7 (2439/7034) |  | 30.8 (1515/4917) | 31.4 (1159/3688) |
| A level |  | 21.7 (2161/9959) | 22.9 (1612/7034) |  | 26.1 (1284/4917) | 28.4 (1046/3688) |
| Degree |  | 12.9 (1289/9959) | 13 (915/7034) |  | 19.9 (977/4917) | 21.1 (780/3688) |
| Birthweight child in grams | 4.4 | 3412.03 (5.5) | 3416.83 (5.58) | 5.3 | 3416.03 (7.67) | 3419.3 (7.84) |
| Gestational age in weeks at delivery | 3.3 | 39.48 (0.02) | 39.49 (0.02) | 4.3 | 39.47 (0.03) | 39.48 (0.03) |
| Maternal pre-pregnancy weight (Kg) | 13.2 | 61.94 (0.11) | 61.92 (0.12) | 11.7 | 61.69 (0.15) | 61.63 (0.16) |
| Maternal pre-pregnancy BMI | 14 | 23.05 (0.04) | 23.04 (0.04) | 12.5 | 22.86 (0.05) | 22.83 (0.06) |
| Maternal smoking during the 1st trimester of pregnancy | 5.8 | 24.7 (2460/9959) | 22.8 (2142/9384) | 5.9 | 17.1 (840/4917) | 16.1 (747/4627) |
| Maternal smoking during the 2nd trimester of pregnancy | 5.8 | 20 (1996/9959) | 18.3 (1716/9384) | 5.9 | 13.5 (666/4917) | 12.7 (589/4627) |
| Maternal smoking during the 3rd trimester of pregnancy (prospectively reported) | 15.8 | 21.4 (2128/9959) | 19.4 (1630/8383) | 14.7 | 13.8 (679/4917) | 13.5 (568/4196) |
| Maternal smoking during the 3rd trimester of pregnancy (retrospectively reported) | 9.5 | 21.1 (2097/9959) | 18.5 (1671/9012) | 8.6 | 13.8 (678/4917) | 12.6 (568/4494) |
| Mother became homeless during pregnancy | 11.9 | 3 (296/9959) | 2.1 (180/8771) | n/a | n/a | n/a |
| Mother divorced since pregnancy | 13.8 | 4.7 (465/9959) | 3.3 (287/8580) | n/a | n/a | n/a |
| Partner hard drug use during pregnancy | 29.1 | 4.1 (411/9959) | 1.7 (121/7061) | n/a | n/a | n/a |
| Mother's partner was emotionally cruel when child was 18yrs | 65.9 | 16.4 (1637/9959) | 4.4 (149/3396) | 41.4 | 11 (539/4917) | 4.1 (117/2879) |
| Antidepressant use by mother when child was 18yrs | 67.5 | 23.7 (2358/9959) | 11.2 (364/3240) | 44.1 | 16.6 (817/4917) | 10.3 (284/2750) |
| Mother separated from partner when child was 18yrs | 65.9 | 18 (1789/9959) | 4.6 (158/3400) | 41.4 | 11.2 (553/4917) | 3.9 (112/2883) |
| Partner of child used physical force when child was 18-21yrs | 72.9 | 52.6 (5236/9959) | 19.3 (520/2697) | 51.2 | 46.4 (2281/4917) | 18 (431/2398) |
| Partner of child used more severe physical force when child was 18-21yrs | 72.9 | 47.4 (4723/9959) | 13 (352/2698) | 51.3 | 41.8 (2054/4917) | 11.8 (283/2395) |
| Partner of child have pressured them into kissing/touching when child was 18-21yrs | 72.9 | 47 (4681/9959) | 14.1 (381/2695) | 51.2 | 42.2 (2075/4917) | 13.2 (318/2400) |
| Partner of child physically forced them into kissing/touching when child was 18-21yrs | 73 | 46 (4582/9959) | 11.3 (304/2693) | 51.3 | 40.4 (1986/4917) | 10.2 (244/2393) |
| Partner of child used pressured them into sexual intercourse when child was 18-21yrs | 73 | 47.6 (4736/9959) | 15.1 (406/2689) | 51.4 | 43.7 (2146/4917) | 14.3 (342/2392) |
| Partner of child physically forced them into sexual intercourse when child was 18-21yrs | 73.1 | 45.7 (4555/9959) | 10.8 (290/2683) | 51.5 | 41.1 (2023/4917) | 10 (238/2386) |
| Partner of child made them feel scared of frightened when child was 18-21yrs | 73.2 | 49.3 (4912/9959) | 19.1 (509/2667) | 51.7 | 45.6 (2241/4917) | 17.9 (426/2376) |
| Difficulty affording food during pregnancy | 10.4 |  |  | n/a | n/a | n/a |
| Not difficult |  | 73.7 (7342/9959) | 76.5 (6822/8921) | n/a | n/a | n/a |
| Some difficulty |  | 16.4 (1629/9959) | 15.6 (1395/8921) | n/a | n/a | n/a |
| Fairly difficult |  | 8 (802/9959) | 6.7 (595/8921) | n/a | n/a | n/a |
| Very difficult |  | 1.9 (187/9959) | 1.2 (109/8921) | n/a | n/a | n/a |
| Difficulty affording heating during pregnancy | 10.4 |  |  | n/a | n/a | n/a |
| Not difficult |  | 69.5 (6918/9959) | 72.4 (6457/8921) | n/a | n/a | n/a |
| Some difficulty |  | 17.7 (1764/9959) | 17.3 (1540/8921) | n/a | n/a | n/a |
| Fairly difficult |  | 9.1 (908/9959) | 7.7 (689/8921) | n/a | n/a | n/a |
| Very difficult |  | 3.7 (369/9959) | 2.6 (235/8921) | n/a | n/a | n/a |
| Mother's opinion of neighbourhood during pregnancy | 8.2 |  |  | n/a | n/a | n/a |
| Very good area |  | 41 (4084/9959) | 41.7 (3818/9147) | n/a | n/a | n/a |
| Fairly good area |  | 51.2 (5096/9959) | 51.3 (4694/9147) | n/a | n/a | n/a |
| Not very good area |  | 5.8 (581/9959) | 5.3 (488/9147) | n/a | n/a | n/a |
| Bad area |  | 2 (198/9959) | 1.6 (147/9147) | n/a | n/a | n/a |
| Maternal depression score (EPDS) when child was 18yrs | 66.2 | 8.92 (0.06) | 7.52 (0.09) | 42.1 | 7.91 (0.08) | 7.25 (0.1) |
| Maternal AUDIT score when child was 18yrs | 71.5 | 8.63 (0.04) | 8.01 (0.06) | 50 | 8.34 (0.05) | 8.04 (0.06) |
| Paternal AUDIT score when child was 18yrs | 84.7 | 9.75 (0.04) | 9.13 (0.08) | 69.1 | 9.38 (0.05) | 9.13 (0.08) |
| Key stage 1: School year taken | 12.4 |  |  | n/a | n/a | n/a |
| 1997 / 1998 |  | 21.1 (2103/9959) | 20.9 (1820/8721) | n/a | n/a | n/a |
| 1998 / 1999 |  | 60.7 (6048/9959) | 60.8 (5303/8721) | n/a | n/a | n/a |
| 1999 / 2000 |  | 18.2 (1808/9959) | 18.3 (1598/8721) | n/a | n/a | n/a |
| Key stage 1: Summary score (prorated) | 12.7 | 9.62 (0.04) | 9.53 (0.04) | n/a | n/a | n/a |
| Key Stage 2: Total marks achieved in English test (sum of reading and writing tests) | 4.4 | 58.15 (0.16) | 58.98 (0.16) | n/a | n/a | n/a |
| Key Stage 2: Total marks achieved in Maths test (sum of Paper A, Paper B and mental arithmetic tests) | 4.1 | 64.44 (0.22) | 65.42 (0.21) | n/a | n/a | n/a |
| Key Stage 2: Total marks achieved in Science test (sum of Paper A and Paper B tests) | 3.2 | 59.07 (0.12) | 59.48 (0.12) | n/a | n/a | n/a |
| Key Stage 2: Total point score as used in the valued added calculations | 82.1 | 84.86 (0.13) | 86.49 (0.31) | n/a | n/a | n/a |
| Key Stage 3: Total marks achieved in English test (sum of reading and writing tests) | 14.4 | 45.71 (0.18) | 46.84 (0.19) | n/a | n/a | n/a |
| Key Stage 3: Total marks achieved in Maths test (sum of Paper A, Paper B and mental arithmetic tests) | 12.3 | 81.91 (0.23) | 82.7 (0.23) | n/a | n/a | n/a |
| Key Stage 3: Total marks achieved in Science test (sum of Paper A and Paper B tests) | 11.8 | 97.24 (0.26) | 98.27 (0.26) | n/a | n/a | n/a |
| Key Stage 3: Total point score as used in the valued added calculations | 9.3 | 106.16 (0.24) | 105.57 (0.25) | n/a | n/a | n/a |
| Key Stage 4: Deprivation Indicator - IDACI score (as used in CVA Model) | 1.5 | 0.15 (0) | 0.15 (0) | n/a | n/a | n/a |
| Key Stage 4: Total GCSE and equivalents new style point score | 0.7 | 407.21 (1.5) | 409.18 (1.49) | n/a | n/a | n/a |
| Key Stage 4: Total GCSE/GNVQ new style point score | 0.2 | 357.83 (1.49) | 357.93 (1.49) | n/a | n/a | n/a |
| Key Stage 4: Number of Full GCSE qualifications at grades A*-C (GCSE equivalencies) | 0.2 | 5.61 (0.04) | 5.62 (0.04) | n/a | n/a | n/a |
| Key Stage 4: Number of Full GCSE qualifications at grades A*-G (GCSE equivalencies) | 0.2 | 7.86 (0.02) | 7.86 (0.02) | n/a | n/a | n/a |
| Key Stage 4: Is pupil known to be eligible for FSM? | 5.6 | 5.2 (515/9959) | 5.1 (479/9398) | n/a | n/a | n/a |
| Key Stage 4: Does pupil have SEN - Action Plus? | 7.4 | 5.6 (553/9959) | 4.8 (444/9225) | n/a | n/a | n/a |
| Key Stage 4: Does pupil have SEN - school action? | 7.4 | 8.2 (819/9959) | 8 (742/9225) | n/a | n/a | n/a |
| >=5 GCSEs including math and English at grades A*-G | 0.2 | 90.6 (9026/9959) | 90.7 (9019/9944) | n/a | n/a | n/a |
| Key Stage 4: Achieved at least 1 GCSE or equivalent at grade A*-G | 0.2 | 98.4 (9802/9959) | 98.4 (9789/9944) | n/a | n/a | n/a |
| Key Stage 4: Achieved 5 or more GCSE/GNVQs at grades A*-C | 0.2 | 63.7 (6341/9959) | 63.7 (6335/9944) | n/a | n/a | n/a |
| Key Stage 4: Achieved 5 or more GCSE/GNVQs at grades A*-G | 0.2 | 92 (9161/9959) | 92.1 (9154/9944) | n/a | n/a | n/a |
| Participating at A levels (Key Stage 5) | 0 | 58.5 (5830-9959) | 58.5 (5830-9959) | n/a | n/a | n/a |
| Capped GCSE and equivalents new style point | 0.9 | 322.13 (0.95) | 324.52 (0.92) | n/a | n/a | n/a |
| Key Stage 5: Student achieved equivalent of 2 A levels | 41.5 | 75.3 (7503/9959) | 95.6 (5566/5824) | n/a | n/a | n/a |
| Key Stage 5: Total re-scaled point score of candidate's entries | 55 | 250.48 (1.7) | 343.69 (2.42) | n/a | n/a | n/a |
| Key Stage 5: Total GCE A Level and equivalent points score based on new QCA points | 41.5 | 611.33 (2.95) | 752.97 (3.3) | n/a | n/a | n/a |
| Key Stage 5: Total number of GCE/VCE A/AS Level & GCE AS/VCE Double Award Level passes (A Levels) | 45.1 | 1.91 (0.02) | 2.8 (0.02) | n/a | n/a | n/a |
| BMI at age 9 | n/a | n/a | n/a | 47.7 | 17.52 (0.04) | 17.43 (0.06) |
| AUDIT score at age 16 | n/a | n/a | n/a | 34.9 | 6.98 (0.08) | 6.59 (0.09) |
| AUDIT score at age 18 | n/a | n/a | n/a | 52.3 | 10.12 (0.09) | 9.1 (0.12) |
| MFQ score at age 10.5 | n/a | n/a | n/a | 12 | 4.05 (0.05) | 3.97 (0.05) |
| MFQ score at age 12.5 | n/a | n/a | n/a | 13.4 | 4.13 (0.06) | 4.02 (0.06) |
| MFQ score at age 16 | n/a | n/a | n/a | 30.2 | 6.04 (0.08) | 5.8 (0.09) |
| MFQ score at age 17 | n/a | n/a | n/a | 14.8 | 6.67 (0.08) | 6.56 (0.08) |
| MFQ score at age 18 | n/a | n/a | n/a | 49.6 | 6.89 (0.09) | 6.62 (0.12) |
| MFQ score at age 16 mum | n/a | n/a | n/a | 28.2 | 2.28 (0.05) | 2.05 (0.05) |
| Maternal smoking at age 17y | n/a | n/a | n/a | 41.2 | 16.8 (828/4917) | 9.5 (274/2889) |
| Smoker at age 13 | n/a | n/a | n/a | 15.8 | 4.2 (207/4917) | 1.7 (71/4141) |
| Smoker at age 15.5 | n/a | n/a | n/a | 19.4 | 12 (592/4917) | 8.5 (338/3964) |
| Smoker at age 16 | n/a | n/a | n/a | 29.3 | 18.2 (894/4917) | 11.2 (388/3475) |
| Smoker at age 18 | n/a | n/a | n/a | 49.4 | 32.2 (1586/4917) | 14.6 (363/2488) |
| Alcohol use at age 13 | n/a | n/a | n/a | 32.3 | 8.3 (411/4917) | 4.9 (162/3329) |
| Illicit drug use at age 13 | n/a | n/a | n/a | 13 | 3.4 (168/4917) | 1.8 (79/4276) |
| Illicit drug use at age 14 | n/a | n/a | n/a | 16 | 7.4 (362/4917) | 4.3 (176/4128) |
| Illicit drug use at age 15.5 | n/a | n/a | n/a | 25.1 | 16 (785/4917) | 12.4 (458/3684) |
| Illicit drug use at age 20 | n/a | n/a | n/a | 41.5 | 25.2 (1238/4917) | 19.8 (569/2878) |
| Illicit drug use at age 16 | n/a | n/a | n/a | 33 | 22 (1082/4917) | 15.2 (502/3292) |
| Illicit drug use at age 18 | n/a | n/a | n/a | 50.4 | 27.5 (1354/4917) | 11.7 (286/2440) |
| Cannabis use at age 15.5 | n/a | n/a | n/a | 20.6 | 5.5 (269/4917) | 2.6 (102/3906) |
| Cannabis use at age 16 | n/a | n/a | n/a | 29.5 | 8.9 (439/4917) | 3 (103/3466) |
| Cannabis use at age 18 | n/a | n/a | n/a | 50.9 | 19.9 (978/4917) | 2.9 (70/2413) |
| Cannabis use at age 20 | n/a | n/a | n/a | 40.9 | 12.3 (606/4917) | 5.1 (149/2904) |

GCSE - General Certificate of Secondary Education; AUDIT - alcohol use disorders identification test; EPDS – Edinburgh Postnatal Depression Scale; IDACI – Income Deprivation Affecting Children Index; CVA – contextual value added; FSM – free school meals; SEN – special educational  needs; GNVQ - General National Vocational Qualification.
