# Supplementary material for: Associations of adverse childhood experiences with educational attainment and adolescent health and the role of family and socioeconomic factors: A prospective cohort study in the UK
Source: PLoS Med. 2020 Mar 2;17(3):e1003031. doi: 10.1371/journal.pmed.1003031 (PMC7051040; doi:10.1371/journal.pmed.1003031)
Supplement: S4 Table — ACE, adverse childhood experience; AUDIT, alcohol use disorders identification test; GCSE, General Certificate of Secondary Education. (DOCX) [file pmed.1003031.s009.docx]

*S4 Table Distributions of outcome and ACE variables in the imputation datasets and in observed data (i.e. without imputation) in boys and girls.*

|  | **Analysis 1: Education** | | | | | | | **Analysis 2: Health** | | | | | | |
| --- | --- | --- | --- | --- | --- | --- | --- | --- | --- | --- | --- | --- | --- | --- |
| **Variable** | **Boys** | | | **Girls** | | | **p-value**Sex difference imputed education data | **Boys** | | | **Girls** | | | **p-value**Sex difference imputed health data |
|  | **% data imputed** | **Distribution**  Mean (SE) for continuous variables   % for categorical variables   In | | **% data imputed** | **Distribution**  Mean (SE) for continuous variables   % for categorical variables   In | |  | **% data imputed** | **Distribution**  Mean (SE) for continuous variables   % for categorical variables   In | | **% data imputed** | **Distribution**  Mean (SE) for continuous variables   % for categorical variables   In | |  |
|  |  | imputed | Observed |  | imputed | Observed |  |  | Imputed | observed |  | imputed | observed |  |
| **OUTCOME** | | | | | | | | | | | | | | |
| <5 GCSEs including math and English at grades A*-C | 0.2 | 50.7 (2553/5032) | 50.6 (2553/5032) | 0.1 | 40.3  (1992/4936) | 40.3 (1992/4936) | <0.01 | n/a | n/a | n/a | n/a | n/a | n/a | n/a |
| BMI-Z at age 17 | n/a | n/a | n/a | n/a | n/a | n/a | n/a | 1.8 | 0.38 (0.03) | 0.38 (0.03) | 1.8 | 0.4 (0.02) | 0.4 (0.02) | 0.68 |
| AUDIT score at age 17 | n/a | n/a | n/a | n/a | n/a | n/a | n/a | 19.8 | 8.64 (0.13) | 8.43 (0.14) | 19.9 | 8.12 (0.11) | 7.93 (0.12) | <0.01 |
| Obesity at age 17 | n/a | n/a | n/a | n/a | n/a | n/a | n/a | 1.8 | 6.3 (136/2163) | 6.3 (134/2125) | 1.8 | 8.0 (221/2754) | 8.0 (216/2704) | 0.02 |
| Depression at age 17 | n/a | n/a | n/a | n/a | n/a | n/a | n/a | 10.6 | 5.5 (118/2163) | 4.5 (87/1933) | 10.6 | 11.1 (307/2754) | 10.6 (262/2462) | <0.01 |
| Smoking at age 17 | n/a | n/a | n/a | n/a | n/a | n/a | n/a | 17.2 | 18.6 (401/2163) | 16.0 (286/1791) | 17.6 | 20.2 (555/2754) | 17.7 (402/2270) | 0.21 |
| Illicit drug use at age 17 | n/a | n/a | n/a | n/a | n/a | n/a | n/a | 18.8 | 17.9 387/2163) | 15.2 (267/1756) | 19.3 | 14.6 (406/2754) | 12.5 (278/2222) | <0.01 |
| Harmful alcohol use at age 17 | n/a | n/a | n/a | n/a | n/a | n/a | n/a | 19.8 | 11.7 (253/2163) | 10.7 (185/1734) | 19.9 | 10.3 (286/2754) | 9.5 (209/2205) | 0.19 |
| **ADVERSE CHILDHOOD EXPERIENCES** | | | | | | | | | | | | | | |
| Categorical ACE-score 0 | 71.6 | 15.5 (781/5032) | 20.7 (295/1428) | 67.0 | 16.7 (1605/4936) | 23.4 (381/1628) | 0.32 | 43.8 | 17.3 (375/2163) | 20.4 (248/1215) | 44.6 | 18.8 (518/2754) | 23.9 (365/1526) | 0.21 |
| 1 |  | 24.4 (1225/5032) | 30 (429/1428) |  | 22.8 (2350/4936) | 26.4 (429/1628) |  |  | 26.9 (581/2163) | 30.8 (374/1215) |  | 24.4 (678/2754) | 27.9 (425/1526) |  |
| 2 3 |  | 36.3 (1824/5032) | 34.7 (495/1428) |  | 36.7 (3634/4936) | 36.3 (591/1628) |  |  | 35.3 (758/2163) | 34.5 (419/1215) |  | 36.7 (990/2754) | 35.8 (547/1526) |  |
| 4+ |  | 23.8 (1194/5032) | 14.6 (209/1428) |  | 23.8 (2370/4936) | 13.9 (227/1628) |  |  | 20.5 (449/2163) | 14.3 (174/1215) |  | 20.1 (568/2754) | 12.4 (189/1526) |  |
| physical abuse | 48.8 | 17.0 (856/5032) | 13.2 (340/2573) | 44.0 | 20.9 (1031/4936) | 16.6 (460/2766) | <0.01 | 28.6 | 20.9 (467/2163) | 17.2 (265/1544) | 26.8 | 20.7 (579/2754) | 17.5 (353/2016) | 0.9 |
| sexual abuse | 24.9 | 2.3 (116/5032) | 0.9 (33/3774) | 23.0 | 6 (295/4936) | 4.7 (177/3802) | <0.01 | 11.7 | 2.4 (53/2163) | 1.3 (25/1911) | 12.3 | 7.0 (195/2754) | 5.9 (142/2414) | <0.01 |
| emotional abuse | 44.0 | 24.0 (1208/5032) | 18.8 (529/2814) | 40.8 | 23.7 (1171/4936) | 19.5 (571/2921) | 0.8 | 25.7 | 22.2 (491/2163) | 17.8 (287/1608) | 25.7 | 23.3 (648/2754) | 19.9 (406/2045) | 0.5 |
| emotional neglect | 54.7 | 26.5 (1333/5032) | 21.4 (486/2276) | 47.1 | 21.2 (1049/4936) | 17.6 (459/2611) | <0.01 | 16.6 | 21.4 (462/2163) | 20.0 (361/1804) | 17.6 | 18.6 (514/2754) | 16.8 (382/2269) | 0.04 |
| bullying | 42.0 | 28.6 (1439/5032) | 27.2 (793/2911) | 36.9 | 23.7 (1169/4936) | 21.3 (665/3117) | <0.01 | 9.7 | 31.0 (673/2163) | 30.4 (593/1953) | 11.8 | 23.6 (653/2754) | 22.6 (548/2429) | <0.01 |
| violence between parents | 47.4 | 25.0 (1254/5032) | 18.8 (498/2644) | 46.3 | 25.7 (1271/4936) | 19.7 (522/2650) | 0.52 | 26.5 | 20.8 (452/2163) | 16.4 (261/1590) | 30.1 | 22.3 (634/2754) | 17.7 (341/1925) | 0.35 |
| substance household | 38.9 | 15.2 (763/5032) | 9.9 (304/3071) | 38.6 | 15.0 (738/4936) | 9.1 (277/3029) | 0.85 | 21.8 | 12.1 (268/2163) | 8.3 (141/1691) | 25.6 | 12.3 (350/2754) | 8.5 (174/2048) | 0.87 |
| mental health problems or suicide | 39.3 | 47.9 (2407/5032) | 42.7 (1300/3047) | 37.7 | 49.3 (2433/4936) | 44.2 (1358/3073) | 0.29 | 20.3 | 43.7 (943/2163) | 40.0 (689/1723) | 23.0 | 45.7 (1263/2754) | 42.1 (892/2120) | 0.23 |
| parent convicted offence | 36.6 | 10.2 (511/5032) | 6.8 (215/3185) | 36.5 | 10.9 (539/4936) | 7.6 (237/3136) | 0.47 | 19.7 | 9.3 (203/2163) | 6.6 (114/1736) | 23.0 | 9.1 (262/2754) | 6.9 (146/2121) | 0.91 |
| parental separation | 46.1 | 33.0 (1658/5032) | 24.3 (659/2708) | 44.4 | 34.7 (1712/4936) | 27.2 (748/2746) | 0.23 | 26.3 | 27.3 (596/2163) | 21.3 (340/1594) | 29.0 | 29.0 (811/2754) | 24.1 (471/1956) | 0.3 |

GCSE - General Certificate of Secondary Education; AUDIT - alcohol use disorders identification test.
