# Supplementary material for: Associations of adverse childhood experiences with educational attainment and adolescent health and the role of family and socioeconomic factors: A prospective cohort study in the UK
Source: PLoS Med. 2020 Mar 2;17(3):e1003031. doi: 10.1371/journal.pmed.1003031 (PMC7051040; doi:10.1371/journal.pmed.1003031)
Supplement: S5 Table — Most variables were part of both the educational attainment as well as the health outcome imputation model, but outcome variables and certain auxiliary variables were specific to one of the two analyses (final column). 1The passive imputation of the ACE score was performed according to I(emotional_abuse + physical_abuse + sexual_abuse + mental_suicide_household + parental_separation + emotional_neglect+ violence_household + bullying + substance_household+ parent_convicted). 2None of the passively imputed variables (ACE count score variable, obesity and harmful alcohol use) were used as a predictor of missingness for the other variables in the imputation model. ACE, adverse childhood experience; AUDIT, alcohol use disorders identification test; CVA, contextual value added; EPDS, Edinburgh Postnatal Depression Scale; FSM, free school meal; GCSE, General Certificate of Secondary Education; GNVQ, General National Vocational Qualification; IDACI, Income Deprivation Affecting Children Index; SEN, special educational needs. (DOCX) [file pmed.1003031.s010.docx]

*S5 Table Description of the variables in the imputation model. Most variables were part of both the educational attainment as well as the health outcome imputation model, but outcome variables and certain auxiliary variables were specific to one of the two analyses (final column).*

| **Variable** | **Type of variable** | **Regression model to predict missing in this variable** | **How variable was entered when used to predict missing in other variables** | **Analysis variable was in the imputation model** |
| --- | --- | --- | --- | --- |
| **OUTCOME** | | | | |
| <5 GCSEs including math and English at grades A*-C | dichotomous | Logistic regression | dichotomous | Education |
| BMI-Z at age 17 | continuous | Predictive mean matching | continuous | Health |
| Obesity at age 17 | dichotomous | Passive imputation to split the BMI-Z score based on IOTF cut-offs for boys (>=2.288) and girls (>=2.192) | dichotomous | Health |
| Depression at age 17 | dichotomous | Logistic regression | dichotomous | Health |
| Smoking at age 17 | dichotomous | Logistic regression | dichotomous | Health |
| Illicit drug use at age 17 | dichotomous | Logistic regression | dichotomous | Health |
| AUDIT score at age 17 | continuous | Predictive mean matching | continuous | Health |
| Harmful alcohol use at age 17 | dichotomous | Passive imputation to split the AUDIT score (>=16) | dichotomous | Health |
| **ADVERSE CHILDHOOD EXPERIENCES** | | | | |
| Categorical ACE-score | categorical (4) | After passive imputation split the sum of 10 ACEs into 0,1,2-3 and 4+ ACEs^1^ | n/a^2^ | Both |
| physical abuse | dichotomous | Logistic regression | dichotomous | Both |
| sexual abuse | dichotomous | Logistic regression | dichotomous | Both |
| emotional abuse | dichotomous | Logistic regression | dichotomous | Both |
| emotional neglect | dichotomous | Logistic regression | dichotomous | Both |
| bullying | dichotomous | Logistic regression | dichotomous | Both |
| violence between parents | dichotomous | Logistic regression | dichotomous | Both |
| substance household | dichotomous | Logistic regression | dichotomous | Both |
| mental health problems or suicide | dichotomous | Logistic regression | dichotomous | Both |
| parent convicted offence | dichotomous | Logistic regression | dichotomous | Both |
| parental separation | dichotomous | Logistic regression | dichotomous | Both |
| **COVARIATES** | | | | |
| Sex | categorical (2) | n/a | categorical (2) | Both |
| Household social class at 18wks gestation | categorical (6) | Polytomous (unordered) regression | 5 indicator variables | Both |
| Ethnicity child | categorical (2) | Logistic regression | 1 indicator variables | Both |
| Maternal age in years at delivery | continuous | Predictive mean matching | continuous | Both |
| Home ownership mother during pregnancy | categorical (7) | Polytomous (unordered) regression | 6 indicator variables | Both |
| Marital status mother during pregnancy | categorical (6) | Polytomous (unordered) regression | 5 indicator variables | Both |
| Parity | continuous | Predictive mean matching | continuous | Both |
| Self-reported highest educational level mother | categorical (5) | Polytomous (unordered) regression | 4 indicator variables | Both |
| Mother-reported highest educational level partner | categorical (5) | Polytomous (unordered) regression | 4 indicator variables | Both |
| Maternal depression score (EPDS) at 18 wks gestation | continuous | Predictive mean matching | continuous | Both |
| Maternal depression score (EPDS) at 32 wks gestation | continuous | Predictive mean matching | continuous | Both |
| Partner depression score (EPDS) at 18 wks gestation | continuous | Predictive mean matching | continuous | Both |
| **AUXILIARY IMPUTATION** | | | | |
| social class | dichotomous | Logistic regression | dichotomous | Both |
| financial difficulties | dichotomous | Logistic regression | dichotomous | Both |
| satisfaction with neighbourhood | dichotomous | Logistic regression | dichotomous | Both |
| social support of child | dichotomous | Logistic regression | dichotomous | Both |
| social support of parent | dichotomous | Logistic regression | dichotomous | Both |
| violence between child and partner | dichotomous | Logistic regression | dichotomous | Both |
| physical illness of the child | dichotomous | Logistic regression | dichotomous | Both |
| physical illness of a parent | dichotomous | Logistic regression | dichotomous | Both |
| parent-child bond | dichotomous | Logistic regression | dichotomous | Both |
| Birthweight child in grams | continuous | Predictive mean matching | continuous | Both |
| Gestational age in weeks at delivery | continuous | Predictive mean matching | continuous | Both |
| Maternal pre-pregnancy weight (Kg) | continuous | Predictive mean matching | continuous | Both |
| Maternal pre-pregnancy BMI | continuous | Predictive mean matching | continuous | Both |
| Self-reported highest educational level partner | categorical (5) | Polytomous (unordered) regression | 4 indicator variables | Both |
| Partner-reported highest educational level mother | categorical (5) | Polytomous (unordered) regression | 4 indicator variables | Both |
| Mother s partner was emotionally cruel when child was 18yrs | dichotomous | Logistic regression | dichotomous | Both |
| Antidepressant use by mother when child was 18yrs | dichotomous | Logistic regression | dichotomous | Both |
| Maternal depression score (EPDS) when child was 18yrs | continuous | Predictive mean matching | continuous | Both |
| Mother separated from partner when child was 18yrs | dichotomous | Logistic regression | dichotomous | Both |
| Maternal AUDIT score when child was 18yrs | continuous | Predictive mean matching | continuous | Both |
| Paternal AUDIT score when child was 18yrs | continuous | Predictive mean matching | continuous | Both |
| Partner of child used physical force when child was 18-21yrs | dichotomous | Logistic regression | dichotomous | Both |
| Partner of child used more severe physical force when child was 18-21yrs | dichotomous | Logistic regression | dichotomous | Both |
| Partner of child have pressured them into kissing/touching when child was 18-21yrs | dichotomous | Logistic regression | dichotomous | Both |
| Partner of child physically forced them into kissing/touching when child was 18-21yrs | dichotomous | Logistic regression | dichotomous | Both |
| Partner of child used pressured them into sexual intercourse when child was 18-21yrs | dichotomous | Logistic regression | dichotomous | Both |
| Partner of child physically forced them into sexual intercourse when child was 18-21yrs | dichotomous | Logistic regression | dichotomous | Both |
| Partner of child made them feel scared of frightened when child was 18-21yrs | dichotomous | Logistic regression | dichotomous | Both |
| Maternal smoking during the 1st trimester of pregnancy | dichotomous | Logistic regression | dichotomous | Both |
| Maternal smoking during the 2nd trimester of pregnancy | dichotomous | Logistic regression | dichotomous | Both |
| Maternal smoking during the 3rd trimester of pregnancy (prospectively reported) | dichotomous | Logistic regression | dichotomous | Both |
| Maternal smoking during the 3rd trimester of pregnancy (retrospectively reported) | dichotomous | Logistic regression | dichotomous | Both |
| Mother became homeless during pregnancy | categorical (2) | Logistic regression | 1 indicator variables | Education |
| Difficulty affording food during pregnancy | categorical (4) | Polytomous (unordered) regression | 3 indicator variables | Education |
| Difficulty affording heating during pregnancy | categorical (4) | Polytomous (unordered) regression | 3 indicator variables | Education |
| Mother’s opinion of neighbourhood during pregnancy | categorical (4) | Polytomous (unordered) regression | 3 indicator variables | Education |
| Mother divorced since pregnancy | dichotomous | Logistic regression | dichotomous | Education |
| Partner hard drug use during pregnancy | categorical (2) | Logistic regression | 1 indicator variables | Education |
| Key stage 1: School year taken | categorical (3) | Polytomous (unordered) regression | 2 indicator variables | Education |
| Key stage 1: Summary score (prorated) | continuous | Predictive mean matching | continuous | Education |
| Key Stage 2: Total marks achieved in English test (sum of reading and writing tests) | continuous | Predictive mean matching | continuous | Education |
| Key Stage 2: Total marks achieved in Maths test (sum of Paper A, Paper B and mental arithmetic tests) | continuous | Predictive mean matching | continuous | Education |
| Key Stage 2: Total marks achieved in Science test (sum of Paper A and Paper B tests) | continuous | Predictive mean matching | continuous | Education |
| Key Stage 2: Total point score as used in the valued added calculations | continuous | Predictive mean matching | continuous | Education |
| Key Stage 3: Total marks achieved in English test (sum of reading and writing tests) | continuous | Predictive mean matching | continuous | Education |
| Key Stage 3: Total marks achieved in Maths test (sum of Paper A, Paper B and mental arithmetic tests) | continuous | Predictive mean matching | continuous | Education |
| Key Stage 3: Total marks achieved in Science test (sum of Paper A and Paper B tests) | continuous | Predictive mean matching | continuous | Education |
| Key Stage 3: Total point score as used in the valued added calculations | continuous | Predictive mean matching | continuous | Education |
| Key Stage 4: Deprivation Indicator - IDACI score (as used in CVA Model) | continuous | Predictive mean matching | continuous | Education |
| Key Stage 4: Is pupil known to be eligible for FSM? | dichotomous | Logistic regression | dichotomous | Education |
| Key Stage 4: Does pupil have SEN - Action Plus? | dichotomous | Logistic regression | dichotomous | Education |
| Key Stage 4: Does pupil have SEN - school action? | dichotomous | Logistic regression | dichotomous | Education |
| Key Stage 4: Total GCSE and equivalents new style point score | continuous | Predictive mean matching | continuous | Education |
| Key Stage 4: Total GCSE/GNVQ new style point score | continuous | Predictive mean matching | continuous | Education |
| >=5 GCSEs including math and English at grades A*-G | dichotomous | Logistic regression | dichotomous | Education |
| Key Stage 4: Achieved at least 1 GCSE or equivalent at grade A*-G | dichotomous | Logistic regression | dichotomous | Education |
| Key Stage 4: Achieved 5 or more GCSE/GNVQs at grades A*-C | dichotomous | Logistic regression | dichotomous | Education |
| Key Stage 4: Achieved 5 or more GCSE/GNVQs at grades A*-G | dichotomous | Logistic regression | dichotomous | Education |
| Key Stage 4: Number of Full GCSE qualifications at grades A*-C (GCSE equivalencies) | continuous | Predictive mean matching | continuous | Education |
| Key Stage 4: Number of Full GCSE qualifications at grades A*-G (GCSE equivalencies) | continuous | Predictive mean matching | continuous | Education |
| Key Stage 4: Capped GCSE and equivalents new style point | continuous | Predictive mean matching | continuous | Education |
| Key Stage 5: Participating at A levels | dichotomous | n/a | dichotomous | Education |
| Key Stage 5: Student achieved equivalent of 2 A levels | categorical (2) | Logistic regression | 1 indicator variables | Education |
| Key Stage 5: Total re-scaled point score of candidate s entries | continuous | Predictive mean matching | continuous | Education |
| Key Stage 5: Total GCE A Level and equivalent points score based on new QCA points | continuous | Predictive mean matching | continuous | Education |
| Key Stage 5: Total number of GCE/VCE A/AS Level & GCE AS/VCE Double Award Level passes (A Levels) | continuous | Predictive mean matching | continuous | Education |
| BMI at age 9 | continuous | Predictive mean matching | continuous | Health |
| Maternal smoking at age 17y | dichotomous | Logistic regression | dichotomous | Health |
| Smoker at age 13 | dichotomous | Logistic regression | dichotomous | Health |
| Smoker at age 15.5 | dichotomous | Logistic regression | dichotomous | Health |
| Smoker at age 16 | dichotomous | Logistic regression | dichotomous | Health |
| Smoker at age 18 | dichotomous | Logistic regression | dichotomous | Health |
| AUDIT score at age 16 | continuous | Predictive mean matching | continuous | Health |
| AUDIT score at age 18 | continuous | Predictive mean matching | continuous | Health |
| Alcohol use at age 13 | dichotomous | Logistic regression | dichotomous | Health |
| MFQ score at age 10.5 | continuous | Predictive mean matching | continuous | Health |
| MFQ score at age 12.5 | continuous | Predictive mean matching | continuous | Health |
| MFQ score at age 16 | continuous | Predictive mean matching | continuous | Health |
| MFQ score at age 17 | continuous | Predictive mean matching | continuous | Health |
| MFQ score at age 18 | continuous | Predictive mean matching | continuous | Health |
| Maternal MFQ score at age 16 | continuous | Predictive mean matching | continuous | Health |
| Illicit drug use at age 13 | dichotomous | Logistic regression | dichotomous | Health |
| Illicit drug use at age 14 | dichotomous | Logistic regression | dichotomous | Health |
| Illicit drug use at age 15.5 | dichotomous | Logistic regression | dichotomous | Health |
| Illicit drug use at age 20 | dichotomous | Logistic regression | dichotomous | Health |
| Illicit drug use at age 16 | dichotomous | Logistic regression | dichotomous | Health |
| Illicit drug use at age 18 | dichotomous | Logistic regression | dichotomous | Health |
| Cannabis use at age 15.5 | dichotomous | Logistic regression | dichotomous | Health |
| Cannabis use at age 16 | dichotomous | Logistic regression | dichotomous | Health |
| Cannabis use at age 18 | dichotomous | Logistic regression | dichotomous | Health |
| Cannabis use at age 20 | dichotomous | Logistic regression | dichotomous | Health |

^1^Formula used for the passive imputation of the ACE score: ~I(as.integer(emotional_abuse)+as.integer(physical_abuse)+as.integer(sexual_abuse)+as.integer(mental_suicide_household)+as.integer(parental_separation)+as.integer(emotional_neglect)+as.integer(violence_household)+as.integer(bullying)+as.integer(substance_household)+as.integer(parent_convicted))

^2^ None of the passively imputed variables (ACE count score variable, obesity and harmful alcohol use) were used as a predictor of missingness for the other variables in the imputation model.

GCSE - General Certificate of Secondary Education; AUDIT - alcohol use disorders identification test; EPDS – Edinburgh Postnatal Depression Scale; IDACI – Income Deprivation Affecting Children Index; CVA – contextual value added; FSM – free school meals; SEN – special educational  needs; GNVQ - General National Vocational Qualification.
