# Supplementary material for: Associations of adverse childhood experiences with educational attainment and adolescent health and the role of family and socioeconomic factors: A prospective cohort study in the UK
Source: PLoS Med. 2020 Mar 2;17(3):e1003031. doi: 10.1371/journal.pmed.1003031 (PMC7051040; doi:10.1371/journal.pmed.1003031)
Supplement: S6 Table — N = 4,917, using multiply imputed data. (DOCX) [file pmed.1003031.s011.docx]

*S6 Table. Associations between health outcomes*

N=4917, using multiply imputed data

|  | **% (n/N) participants with each outcome who also report:** | | | | |
| --- | --- | --- | --- | --- | --- |
|  | **Smoking** | **Depression** | **Illicit drug use** | **Obese** | **Harmful alcohol abuse** |
| **Smoking** | 100 (958/958) | 15 (140/958) | 45 (428/958) | 10 (93/958) | 26 (249/958) |
| **Depression** | 33 (140/426) | 100 (426/426) | 29 (125/426) | 11 (45/426) | 20 (84/426) |
| **Illicit drug use** | 54 (428/791) | 16 (125/791) | 100 (791/791) | 6 (45/791) | 28 (225/791) |
| **Obese** | 26 (93/357) | 13 (45/357) | 12 (45/357) | 100 (357/357) | 10 (36/357) |
| **Harmful alcohol abuse** | 46 (249/539) | 16 (84/539) | 42 (225/539) | 7 (36/539) | 100 (539/539) |
