# Supplementary material for: Associations of adverse childhood experiences with educational attainment and adolescent health and the role of family and socioeconomic factors: A prospective cohort study in the UK
Source: PLoS Med. 2020 Mar 2;17(3):e1003031. doi: 10.1371/journal.pmed.1003031 (PMC7051040; doi:10.1371/journal.pmed.1003031)
Supplement: S7 Table — The basic model was adjusted for sex, whereas the adjusted model also includes sociodemographic indicators. The p-value of the interaction test is for the comparison of the original model with a model including a sex interaction. Furthermore, model estimates of sex-stratified analyses are given. ACE, adverse childhood experience; AOR, adjusted odds ratio; CI, confidence interval. (DOCX) [file pmed.1003031.s012.docx]

*S7 Table Adjusted odds ratios (AOR), 95% confidence intervals and p-values for the association between the ACE measures and educational attainment or health. The basic model was adjusted for sex, whereas the adjusted model also includes sociodemographic indicators. The p-value of the interaction test is for the comparison of the original model with a model including a sex interaction. Furthermore, model estimates of sex stratified analyses are given.*

| **Adversity** | **Model** | **Analysis 1: Education** | |  | **Analysis 2: Health** | | | | | |
| --- | --- | --- | --- | --- | --- | --- | --- | --- | --- | --- |
|  |  | GCSE score Estimate 95% CI | <5 good GCSE   AOR 95% CI | Depression   AOR 95% CI | | Harmful alcohol use  AOR 95% CI | Illicit drug use AOR 95% CI | BMI -Z Estimate 95% CI | Obesity AOR 95% CI | Smoking AOR 95% CI |
| **Categorical ACE score** | Basic 1 ACE | -15.00 (-22.01, -7.99), p<0.001 | 1.38 (1.17,1.62), p<0.001 | 1.35 (0.87,2.11), p=0.182 | | 1.03 (0.73,1.45), p=0.872 | 1.45 (1.04,2.02), p=0.026 | 0.11 ( 0.00,0.23), p=0.059 | 1.40 (0.92,2.12), p=0.117 | 1.19 (0.89,1.58), p=0.233 |
|  | Basic 2-3 ACEs | -31.49 (-37.79,-25.19), p<0.001 | 1.77 (1.52,2.06), p<0.001 | 2.14 (1.46,3.15), p<0.001 | | 1.32 (0.96,1.80), p=0.084 | 1.88 (1.39,2.56), p<0.001 | 0.15 ( 0.04,0.26), p=0.006 | 1.59 (1.08,2.34), p=0.018 | 1.70 (1.31,2.21), p<0.001 |
|  | Basic 4+ ACEs | -69.38 (-76.40,-62.37), p<0.001 | 3.18 (2.69,3.75), p<0.001 | 3.09 (2.07,4.61), p<0.001 | | 1.57 (1.12,2.20), p=0.008 | 3.35 (2.41,4.67), p<0.001 | 0.19 ( 0.07,0.31), p=0.002 | 1.87 (1.24,2.82), p=0.003 | 2.72 (2.06,3.58), p<0.001 |
|  | Adjusted 1 ACE | -11.99 (-18.02, -5.96), p<0.001 | 1.37 (1.15,1.63), p<0.001 | 1.29 (0.83,2.02), p=0.260 | | 0.98 (0.69,1.40), p=0.931 | 1.44 (1.03,2.01), p=0.033 | 0.10 (-0.02,0.22), p=0.089 | 1.38 (0.90,2.11), p=0.139 | 1.17 (0.88,1.56), p=0.286 |
|  | Adjusted 2-3 ACEs | -21.15 (-26.81,-15.50), p<0.001 | 1.57 (1.33,1.86), p<0.001 | 1.94 (1.31,2.88), p=0.001 | | 1.21 (0.88,1.68), p=0.244 | 1.81 (1.32,2.48), p<0.001 | 0.12 ( 0.01,0.23), p=0.030 | 1.51 (1.02,2.24), p=0.040 | 1.63 (1.24,2.13), p<0.001 |
|  | Adjusted 4+ ACEs | -37.20 (-44.40,-30.00), p<0.001 | 2.00 (1.65,2.43), p<0.001 | 2.43 (1.57,3.77), p<0.001 | | 1.36 (0.94,1.97), p=0.101 | 3.06 (2.13,4.40), p<0.001 | 0.07 (-0.06,0.21), p=0.265 | 1.42 (0.90,2.24), p=0.130 | 2.26 (1.66,3.09), p<0.001 |
|  | Adjusted complete cases 1 ACE | -7.18 (-13.53, -0.82), p=0.027 | 1.21 (0.92,1.61), p=0.182 | 1.01 (0.57,1.82), p=0.961 | | 0.96 (0.60,1.55), p=0.876 | 1.71 (1.09,2.76), p=0.023 | -0.01 (-0.15,0.12), p=0.837 | 0.93 (0.55,1.57), p=0.775 | 1.01 (0.67,1.52), p=0.962 |
|  | Adjusted complete cases 2-3 ACEs | -12.04 (-18.21, -5.87), p<0.001 | 1.25 (0.95,1.64), p=0.107 | 1.92 (1.16,3.25), p=0.013 | | 1.19 (0.76,1.87), p=0.452 | 2.31 (1.50,3.65), p<0.001 | 0.06 (-0.07,0.20), p=0.367 | 1.33 (0.83,2.19), p=0.248 | 1.56 (1.08,2.29), p=0.021 |
|  | Adjusted complete cases 4+ ACEs | -15.23 (-23.22, -7.25), p<0.001 | 1.46 (1.04,2.05), p=0.029 | 2.25 (1.22,4.18), p=0.010 | | 0.97 (0.52,1.77), p=0.933 | 3.45 (2.05,5.86), p<0.001 | 0.00 (-0.18,0.18), p=0.994 | 1.12 (0.58,2.12), p=0.730 | 2.09 (1.31,3.32), p=0.002 |
|  | Sex interaction test | p=0.730 | p=0.928 | p=0.652 | | p=0.296 | p=0.494 | p=0.169 | p=0.188 | p=0.455 |
|  | Sex interaction term 1 ACE | 4.78 ( -7.16, 16.72), p=0.432 | 1.05 (0.74,1.48), p=0.795 | 1.47 (0.54,4.00), p=0.447 | | 0.56 (0.28,1.12), p=0.100 | 1.11 (0.58,2.12), p=0.743 | 0.13 (-0.10,0.36), p=0.258 | 2.44 (1.03,5.81), p=0.043 | 1.14 (0.63,2.07), p=0.664 |
|  | Sex interaction term 2-3 ACEs | 6.19 ( -5.37, 17.74), p=0.294 | 0.98 (0.71,1.37), p=0.920 | 0.99 (0.41,2.37), p=0.981 | | 0.57 (0.30,1.10), p=0.096 | 1.02 (0.55,1.88), p=0.954 | 0.23 ( 0.01,0.44), p=0.036 | 2.12 (0.96,4.66), p=0.062 | 0.98 (0.57,1.67), p=0.940 |
|  | Sex interaction term 4+ ACEs | 7.01 ( -6.03, 20.04), p=0.292 | 1.08 (0.76,1.53), p=0.681 | 1.35 (0.54,3.40), p=0.518 | | 0.79 (0.39,1.58), p=0.498 | 1.42 (0.77,2.65), p=0.263 | 0.24 ( 0.00,0.47), p=0.048 | 2.36 (1.01,5.49), p=0.047 | 1.38 (0.79,2.42), p=0.255 |
|  | Boys 1 ACE | -14.35 (-23.37, -5.34), p=0.002 | 1.35 (1.06,1.72), p=0.017 | 0.98 (0.42,2.29), p=0.957 | | 1.36 (0.80,2.31), p=0.261 | 1.36 (0.84,2.21), p=0.213 | 0.02 (-0.14,0.19), p=0.779 | 0.84 (0.45,1.57), p=0.586 | 1.11 (0.71,1.73), p=0.652 |
|  | Boys 2-3 ACEs | -23.94 (-32.74,-15.14), p<0.001 | 1.59 (1.26,2.01), p<0.001 | 1.86 (0.87,3.98), p=0.110 | | 1.67 (0.99,2.80), p=0.054 | 1.80 (1.14,2.84), p=0.011 | 0.00 (-0.16,0.17), p=0.979 | 1.03 (0.58,1.82), p=0.916 | 1.69 (1.13,2.53), p=0.011 |
|  | Boys 4+ ACEs | -39.94 (-50.84,-29.05), p<0.001 | 1.93 (1.46,2.54), p<0.001 | 1.76 (0.75,4.11), p=0.191 | | 1.63 (0.91,2.92), p=0.098 | 2.70 (1.63,4.50), p<0.001 | -0.01 (-0.20,0.19), p=0.945 | 1.02 (0.51,2.03), p=0.957 | 2.03 (1.30,3.19), p=0.002 |
|  | Girls 1 ACE | -9.83 (-17.80, -1.87), p=0.016 | 1.40 (1.08,1.81), p=0.010 | 1.44 (0.84,2.46), p=0.180 | | 0.75 (0.47,1.21), p=0.243 | 1.51 (0.96,2.38), p=0.072 | 0.15 (-0.01,0.30), p=0.066 | 1.96 (1.07,3.61), p=0.029 | 1.21 (0.82,1.78), p=0.347 |
|  | Girls 2-3 ACEs | -18.64 (-26.21,-11.08), p<0.001 | 1.56 (1.22,2.00), p<0.001 | 1.96 (1.22,3.12), p=0.005 | | 0.95 (0.62,1.47), p=0.824 | 1.81 (1.17,2.80), p=0.007 | 0.21 ( 0.06,0.35), p=0.005 | 2.05 (1.15,3.63), p=0.015 | 1.56 (1.07,2.26), p=0.019 |
|  | Girls 4+ ACEs | -35.06 (-45.07,-25.04), p<0.001 | 2.10 (1.59,2.77), p<0.001 | 2.80 (1.66,4.72), p<0.001 | | 1.18 (0.71,1.95), p=0.521 | 3.42 (2.10,5.57), p<0.001 | 0.14 (-0.04,0.32), p=0.123 | 1.83 (0.95,3.52), p=0.069 | 2.36 (1.54,3.61), p<0.001 |
| **Physical abuse** | Basic | -14.40 (-22.78, -6.01), p<0.001 | 1.15 (0.98,1.34), p=0.085 | 1.97 (1.51,2.56), p<0.001 | | 1.28 (1.00,1.65), p=0.051 | 1.68 (1.35,2.10), p<0.001 | 0.04 (-0.06,0.13), p=0.490 | 1.45 (1.09,1.93), p=0.010 | 1.80 (1.47,2.21), p<0.001 |
|  | Adjusted | -1.68 ( -8.89,  5.53), p=0.647 | 0.91 (0.76,1.07), p=0.255 | 1.85 (1.41,2.43), p<0.001 | | 1.22 (0.94,1.58), p=0.144 | 1.58 (1.25,2.00), p<0.001 | 0.00 (-0.11,0.10), p=0.938 | 1.32 (0.98,1.78), p=0.071 | 1.65 (1.32,2.06), p<0.001 |
|  | Adjusted complete cases | 2.87 ( -3.16,  8.89), p=0.351 | 0.83 (0.67,1.01), p=0.069 | 2.04 (1.41,2.91), p<0.001 | | 1.01 (0.68,1.46), p=0.971 | 1.58 (1.15,2.13), p=0.004 | -0.02 (-0.14,0.10), p=0.716 | 1.33 (0.89,1.95), p=0.148 | 1.67 (1.26,2.21), p<0.001 |
|  | Sex interaction test | p=0.860 | p=1.000 | p=0.431 | | p=0.540 | p=0.304 | p=0.444 | p=0.828 | p=0.062 |
|  | Sex interaction term | 1.21 (-12.28, 14.70), p=0.860 | 1.00 (0.72,1.40), p=0.996 | 1.33 (0.68,2.61), p=0.404 | | 1.18 (0.70,2.01), p=0.538 | 1.26 (0.81,1.97), p=0.310 | 0.08 (-0.12,0.28), p=0.444 | 1.07 (0.58,1.98), p=0.824 | 1.50 (0.98,2.30), p=0.063 |
|  | Boys | -2.27 (-13.17,  8.62), p=0.681 | 0.91 (0.71,1.17), p=0.447 | 1.57 (0.86,2.88), p=0.141 | | 1.11 (0.74,1.67), p=0.607 | 1.47 (1.05,2.05), p=0.024 | -0.02 (-0.18,0.14), p=0.814 | 1.39 (0.85,2.28), p=0.187 | 1.39 (1.01,1.92), p=0.043 |
|  | Girls | -1.57 (-10.81,  7.66), p=0.737 | 0.91 (0.71,1.15), p=0.427 | 2.05 (1.48,2.83), p<0.001 | | 1.29 (0.90,1.85), p=0.158 | 1.73 (1.23,2.42), p=0.002 | 0.01 (-0.13,0.14), p=0.939 | 1.25 (0.84,1.87), p=0.267 | 1.92 (1.41,2.62), p<0.001 |
| **Sexual abuse** | Basic | -26.34 (-39.60,-13.08), p<0.001 | 1.83 (1.40,2.39), p<0.001 | 2.28 (1.53,3.40), p<0.001 | | 1.53 (1.00,2.34), p=0.052 | 1.54 (1.02,2.32), p=0.040 | 0.24 ( 0.05,0.43), p=0.011 | 1.70 (1.06,2.75), p=0.029 | 1.96 (1.39,2.77), p<0.001 |
|  | Adjusted | -4.65 (-16.90,  7.61), p=0.456 | 1.35 (0.99,1.84), p=0.058 | 2.07 (1.36,3.15), p<0.001 | | 1.46 (0.93,2.27), p=0.097 | 1.41 (0.91,2.17), p=0.121 | 0.15 (-0.03,0.34), p=0.108 | 1.34 (0.81,2.20), p=0.257 | 1.60 (1.11,2.29), p=0.011 |
|  | Adjusted complete cases | -7.20 (-20.18,  5.78), p=0.277 | 1.25 (0.83,1.87), p=0.284 | 2.50 (1.38,4.31), p=0.002 | | 2.05 (1.04,3.76), p=0.027 | 1.82 (0.99,3.18), p=0.043 | 0.14 (-0.10,0.38), p=0.261 | 1.19 (0.56,2.28), p=0.624 | 1.82 (1.08,2.99), p=0.020 |
|  | Sex interaction test | p=0.827 | p=0.188 | p=0.491 | | p=0.721 | p=0.131 | p=0.951 | p=0.551 | p=0.878 |
|  | Sex interaction term | 3.12 (-24.86, 31.09), p=0.827 | 0.60 (0.28,1.27), p=0.181 | 0.68 (0.22,2.08), p=0.498 | | 1.23 (0.42,3.58), p=0.705 | 2.32 (0.76,7.09), p=0.138 | -0.01 (-0.46,0.43), p=0.951 | 0.70 (0.22,2.26), p=0.552 | 0.92 (0.37,2.30), p=0.854 |
|  | Boys | -6.26 (-32.49, 19.97), p=0.639 | 1.95 (0.98,3.89), p=0.058 | 3.06 (1.00,9.32), p=0.049 | | 1.24 (0.45,3.40), p=0.678 | 0.78 (0.27,2.25), p=0.645 | 0.25 (-0.16,0.65), p=0.232 | 2.20 (0.73,6.59), p=0.160 | 1.92 (0.85,4.36), p=0.117 |
|  | Girls | -4.70 (-17.39,  7.99), p=0.466 | 1.20 (0.85,1.68), p=0.298 | 1.96 (1.25,3.07), p=0.004 | | 1.49 (0.91,2.44), p=0.109 | 1.72 (1.06,2.78), p=0.029 | 0.13 (-0.09,0.34), p=0.251 | 1.13 (0.63,2.03), p=0.674 | 1.48 (0.97,2.25), p=0.069 |
| **Emotional abuse** | Basic | -20.74 (-27.34,-14.13), p<0.001 | 1.36 (1.20,1.54), p<0.001 | 1.65 (1.27,2.16), p<0.001 | | 1.22 (0.93,1.59), p=0.146 | 1.85 (1.49,2.29), p<0.001 | -0.01 (-0.10,0.08), p=0.827 | 1.12 (0.84,1.51), p=0.441 | 1.45 (1.18,1.79), p<0.001 |
|  | Adjusted | -8.33 (-14.32, -2.35), p=0.007 | 1.14 (0.98,1.31), p=0.084 | 1.42 (1.06,1.90), p=0.018 | | 1.12 (0.84,1.49), p=0.435 | 1.65 (1.31,2.08), p<0.001 | -0.06 (-0.16,0.04), p=0.226 | 1.00 (0.72,1.38), p=0.981 | 1.27 (1.02,1.60), p=0.037 |
|  | Adjusted complete cases | -4.53 ( -9.95,  0.89), p=0.102 | 1.12 (0.94,1.33), p=0.205 | 1.32 (0.90,1.90), p=0.141 | | 0.97 (0.66,1.38), p=0.853 | 1.84 (1.37,2.45), p<0.001 | -0.08 (-0.20,0.03), p=0.160 | 1.01 (0.67,1.49), p=0.966 | 1.32 (0.99,1.74), p=0.052 |
|  | Sex interaction test | p=0.489 | p=0.966 | p=0.399 | | p=0.961 | p=0.801 | p=0.642 | p=0.765 | p=0.886 |
|  | Sex interaction term | 3.90 ( -7.19, 15.00), p=0.489 | 1.01 (0.76,1.33), p=0.966 | 1.34 (0.69,2.58), p=0.385 | | 1.02 (0.60,1.72), p=0.943 | 1.06 (0.68,1.65), p=0.808 | -0.04 (-0.23,0.14), p=0.642 | 0.91 (0.49,1.69), p=0.771 | 1.03 (0.67,1.60), p=0.891 |
|  | Boys | -10.18 (-18.96, -1.40), p=0.023 | 1.14 (0.93,1.40), p=0.207 | 1.07 (0.57,2.02), p=0.838 | | 1.14 (0.73,1.79), p=0.562 | 1.71 (1.18,2.47), p=0.005 | 0.01 (-0.14,0.16), p=0.910 | 1.18 (0.70,1.98), p=0.546 | 1.30 (0.93,1.81), p=0.130 |
|  | Girls | -6.34 (-14.37,  1.69), p=0.121 | 1.15 (0.93,1.42), p=0.205 | 1.57 (1.13,2.18), p=0.007 | | 1.12 (0.78,1.62), p=0.539 | 1.64 (1.19,2.25), p=0.002 | -0.11 (-0.23,0.02), p=0.102 | 0.86 (0.56,1.32), p=0.496 | 1.21 (0.88,1.67), p=0.235 |
| **Emotional neglect** | Basic | -46.35 (-54.52,-38.17), p<0.001 | 2.31 (2.01,2.65), p<0.001 | 1.24 (0.94,1.64), p=0.135 | | 0.96 (0.71,1.29), p=0.783 | 1.12 (0.87,1.44), p=0.376 | 0.16 ( 0.07,0.26), p<0.001 | 1.48 (1.12,1.96), p=0.006 | 1.17 (0.94,1.47), p=0.154 |
|  | Adjusted | -28.96 (-35.94,-21.98), p<0.001 | 1.90 (1.64,2.20), p<0.001 | 1.14 (0.86,1.52), p=0.368 | | 0.94 (0.69,1.28), p=0.706 | 1.09 (0.84,1.42), p=0.496 | 0.11 ( 0.02,0.21), p=0.022 | 1.25 (0.94,1.67), p=0.130 | 1.06 (0.84,1.33), p=0.621 |
|  | Adjusted complete cases | -21.92 (-27.14,-16.70), p<0.001 | 1.81 (1.49,2.20), p<0.001 | 1.31 (0.88,1.90), p=0.167 | | 1.06 (0.71,1.55), p=0.751 | 0.85 (0.59,1.19), p=0.362 | 0.09 (-0.02,0.21), p=0.112 | 1.36 (0.93,1.96), p=0.105 | 0.96 (0.70,1.30), p=0.794 |
|  | Sex interaction test | p=0.035 | p=0.896 | p=0.232 | | p=0.701 | p=0.179 | p=0.169 | p=0.511 | p=0.039 |
|  | Sex interaction term | 16.38 (  1.20, 31.55), p=0.035 | 1.02 (0.75,1.38), p=0.903 | 0.68 (0.36,1.27), p=0.226 | | 0.89 (0.50,1.58), p=0.691 | 1.40 (0.85,2.31), p=0.190 | 0.13 (-0.06,0.32), p=0.169 | 1.22 (0.68,2.19), p=0.513 | 1.63 (1.02,2.60), p=0.040 |
|  | Boys | -35.85 (-46.55,-25.14), p<0.001 | 1.90 (1.54,2.35), p<0.001 | 1.46 (0.87,2.46), p=0.154 | | 1.01 (0.67,1.52), p=0.962 | 0.90 (0.62,1.31), p=0.579 | 0.07 (-0.07,0.21), p=0.311 | 1.25 (0.80,1.95), p=0.331 | 0.79 (0.55,1.14), p=0.209 |
|  | Girls | -21.17 (-31.45,-10.88), p<0.001 | 1.92 (1.54,2.39), p<0.001 | 1.02 (0.70,1.48), p=0.920 | | 0.86 (0.55,1.35), p=0.520 | 1.29 (0.90,1.85), p=0.169 | 0.16 ( 0.03,0.30), p=0.020 | 1.30 (0.88,1.93), p=0.191 | 1.29 (0.96,1.75), p=0.093 |
| **Bullying** | Basic | -21.55 (-28.20,-14.89), p<0.001 | 1.52 (1.35,1.71), p<0.001 | 1.65 (1.30,2.10), p<0.001 | | 1.18 (0.94,1.48), p=0.145 | 1.23 (1.00,1.51), p=0.053 | 0.05 (-0.03,0.14), p=0.186 | 1.27 (0.99,1.63), p=0.059 | 1.26 (1.05,1.50), p=0.011 |
|  | Adjusted | -15.61 (-21.31, -9.92), p<0.001 | 1.48 (1.30,1.69), p<0.001 | 1.55 (1.21,1.98), p<0.001 | | 1.13 (0.90,1.41), p=0.312 | 1.17 (0.94,1.44), p=0.153 | 0.04 (-0.04,0.12), p=0.375 | 1.20 (0.93,1.54), p=0.167 | 1.18 (0.99,1.42), p=0.065 |
|  | Adjusted complete cases | -9.50 (-14.08, -4.91), p<0.001 | 1.35 (1.15,1.59), p<0.001 | 1.41 (1.02,1.95), p=0.037 | | 1.06 (0.76,1.44), p=0.739 | 1.11 (0.84,1.45), p=0.452 | 0.03 (-0.07,0.13), p=0.564 | 1.19 (0.85,1.65), p=0.292 | 1.06 (0.82,1.36), p=0.653 |
|  | Sex interaction test | p=0.153 | p=0.009 | p=0.885 | | p=0.259 | p=0.488 | p=0.645 | p=0.291 | p=0.179 |
|  | Sex interaction term | -8.61 (-20.43,  3.22), p=0.153 | 1.41 (1.10,1.81), p=0.008 | 0.96 (0.57,1.61), p=0.873 | | 1.29 (0.83,2.03), p=0.261 | 0.87 (0.58,1.30), p=0.487 | 0.04 (-0.12,0.20), p=0.646 | 1.32 (0.78,2.24), p=0.296 | 1.29 (0.89,1.86), p=0.175 |
|  | Boys | -11.42 (-20.42, -2.42), p=0.013 | 1.25 (1.05,1.50), p=0.013 | 1.57 (1.00,2.47), p=0.050 | | 0.99 (0.71,1.37), p=0.940 | 1.25 (0.94,1.66), p=0.132 | 0.03 (-0.08,0.14), p=0.594 | 1.02 (0.68,1.53), p=0.919 | 1.02 (0.77,1.35), p=0.887 |
|  | Girls | -20.74 (-28.05,-13.43), p<0.001 | 1.78 (1.48,2.15), p<0.001 | 1.53 (1.13,2.05), p=0.005 | | 1.24 (0.90,1.70), p=0.185 | 1.05 (0.77,1.43), p=0.756 | 0.05 (-0.07,0.16), p=0.426 | 1.31 (0.93,1.84), p=0.129 | 1.27 (0.99,1.62), p=0.059 |
| **Violence between parents** | Basic | -29.87 (-36.75,-22.99), p<0.001 | 1.59 (1.40,1.81), p<0.001 | 1.07 (0.78,1.46), p=0.693 | | 1.24 (0.95,1.61), p=0.111 | 1.76 (1.41,2.20), p<0.001 | 0.10 (-0.01,0.21), p=0.065 | 1.21 (0.89,1.64), p=0.220 | 1.57 (1.27,1.94), p<0.001 |
|  | Adjusted | -9.46 (-16.29, -2.63), p=0.007 | 1.14 (0.98,1.33), p=0.086 | 0.85 (0.60,1.20), p=0.360 | | 1.13 (0.85,1.50), p=0.393 | 1.57 (1.24,2.00), p<0.001 | 0.03 (-0.08,0.15), p=0.537 | 1.04 (0.75,1.44), p=0.815 | 1.36 (1.08,1.71), p=0.010 |
|  | Adjusted complete cases | 0.73 ( -4.79,  6.25), p=0.796 | 0.96 (0.79,1.15), p=0.642 | 1.04 (0.68,1.56), p=0.847 | | 0.98 (0.65,1.45), p=0.938 | 1.65 (1.20,2.24), p=0.002 | 0.06 (-0.06,0.18), p=0.359 | 1.05 (0.67,1.58), p=0.832 | 1.48 (1.10,1.98), p=0.009 |
|  | Sex interaction test | p=0.522 | p=0.536 | p=0.383 | | p=0.697 | p=0.799 | p=0.691 | p=0.752 | p=0.929 |
|  | Sex interaction term | -3.47 (-14.13,  7.19), p=0.522 | 1.09 (0.83,1.42), p=0.535 | 1.47 (0.65,3.33), p=0.357 | | 1.11 (0.65,1.90), p=0.695 | 0.93 (0.58,1.49), p=0.765 | 0.04 (-0.16,0.24), p=0.691 | 1.11 (0.58,2.13), p=0.746 | 0.98 (0.62,1.54), p=0.927 |
|  | Boys | -7.16 (-16.96,  2.64), p=0.151 | 1.09 (0.89,1.33), p=0.398 | 0.59 (0.26,1.33), p=0.199 | | 1.06 (0.70,1.62), p=0.773 | 1.72 (1.20,2.47), p=0.003 | 0.05 (-0.11,0.20), p=0.548 | 1.07 (0.63,1.84), p=0.796 | 1.41 (0.99,2.01), p=0.054 |
|  | Girls | -12.24 (-20.23, -4.24), p=0.003 | 1.20 (0.97,1.50), p=0.092 | 0.96 (0.66,1.40), p=0.835 | | 1.17 (0.80,1.73), p=0.419 | 1.44 (1.02,2.04), p=0.038 | 0.03 (-0.12,0.18), p=0.694 | 1.03 (0.67,1.59), p=0.896 | 1.28 (0.92,1.76), p=0.138 |
| **Substance use household** | Basic | -41.78 (-49.65,-33.90), p<0.001 | 1.72 (1.49,2.00), p<0.001 | 1.29 (0.90,1.86), p=0.171 | | 1.62 (1.21,2.17), p=0.001 | 2.53 (1.94,3.29), p<0.001 | 0.03 (-0.09,0.16), p=0.615 | 1.00 (0.66,1.53), p=0.982 | 1.80 (1.40,2.31), p<0.001 |
|  | Adjusted | -14.13 (-21.58, -6.67), p<0.001 | 1.07 (0.90,1.28), p=0.433 | 0.96 (0.64,1.42), p=0.821 | | 1.48 (1.07,2.05), p=0.017 | 2.14 (1.60,2.88), p<0.001 | -0.05 (-0.19,0.08), p=0.450 | 0.79 (0.50,1.25), p=0.317 | 1.49 (1.12,1.98), p=0.007 |
|  | Adjusted complete cases | -5.72 (-12.96,  1.53), p=0.122 | 0.96 (0.76,1.20), p=0.706 | 1.00 (0.57,1.65), p=0.989 | | 1.66 (1.02,2.60), p=0.034 | 2.16 (1.47,3.13), p<0.001 | -0.09 (-0.26,0.07), p=0.274 | 0.56 (0.26,1.07), p=0.103 | 1.46 (0.98,2.13), p=0.053 |
|  | Sex interaction test | p=0.331 | p=0.733 | p=1.000 | | p=0.572 | p=0.039 | p=0.312 | p=0.313 | p=0.194 |
|  | Sex interaction term | 7.24 ( -7.41, 21.89), p=0.331 | 1.07 (0.74,1.53), p=0.730 | 1.02 (0.45,2.35), p=0.954 | | 1.19 (0.65,2.17), p=0.567 | 1.76 (1.03,3.01), p=0.040 | -0.13 (-0.37,0.12), p=0.312 | 0.64 (0.28,1.49), p=0.304 | 1.45 (0.84,2.51), p=0.181 |
|  | Boys | -17.32 (-28.11, -6.52), p=0.002 | 1.03 (0.80,1.33), p=0.826 | 0.88 (0.40,1.92), p=0.743 | | 1.45 (0.89,2.35), p=0.134 | 1.66 (1.05,2.62), p=0.030 | 0.06 (-0.13,0.26), p=0.519 | 1.11 (0.58,2.13), p=0.752 | 1.28 (0.83,1.98), p=0.261 |
|  | Girls | -11.07 (-22.38,  0.25), p=0.055 | 1.12 (0.85,1.48), p=0.410 | 1.00 (0.63,1.59), p=0.986 | | 1.51 (0.96,2.36), p=0.071 | 2.70 (1.82,4.00), p<0.001 | -0.15 (-0.34,0.04), p=0.116 | 0.58 (0.30,1.13), p=0.110 | 1.70 (1.14,2.54), p=0.010 |
| **Parental mental health problems or suicide** | Basic | -27.88 (-32.86,-22.91), p<0.001 | 1.57 (1.42,1.74), p<0.001 | 1.69 (1.34,2.14), p<0.001 | | 1.14 (0.92,1.40), p=0.233 | 1.53 (1.26,1.85), p<0.001 | 0.08 ( 0.00,0.16), p=0.037 | 1.21 (0.95,1.53), p=0.125 | 1.43 (1.21,1.69), p<0.001 |
|  | Adjusted | -10.29 (-15.19, -5.39), p<0.001 | 1.20 (1.06,1.36), p=0.004 | 1.44 (1.10,1.89), p=0.008 | | 0.98 (0.78,1.24), p=0.878 | 1.41 (1.14,1.75), p=0.001 | 0.02 (-0.06,0.11), p=0.603 | 1.08 (0.82,1.42), p=0.597 | 1.27 (1.05,1.54), p=0.014 |
|  | Adjusted complete cases | -5.90 (-10.30, -1.49), p=0.009 | 1.13 (0.98,1.30), p=0.096 | 1.25 (0.90,1.74), p=0.179 | | 0.80 (0.58,1.09), p=0.165 | 1.42 (1.09,1.85), p=0.010 | -0.03 (-0.13,0.06), p=0.497 | 0.97 (0.69,1.36), p=0.872 | 1.18 (0.92,1.51), p=0.189 |
|  | Sex interaction test | p=0.505 | p=0.838 | p=0.279 | | p=0.376 | p=0.121 | p=0.003 | p=0.255 | p=1.000 |
|  | Sex interaction term | -2.92 (-11.52,  5.68), p=0.505 | 1.02 (0.83,1.27), p=0.839 | 1.35 (0.79,2.31), p=0.275 | | 0.82 (0.54,1.26), p=0.371 | 1.34 (0.92,1.94), p=0.124 | 0.22 ( 0.08,0.37), p=0.003 | 1.34 (0.81,2.21), p=0.258 | 0.99 (0.69,1.41), p=0.962 |
|  | Boys | -8.17 (-15.73, -0.62), p=0.034 | 1.20 (1.01,1.43), p=0.040 | 1.03 (0.60,1.79), p=0.903 | | 1.11 (0.78,1.58), p=0.557 | 1.25 (0.91,1.72), p=0.160 | -0.09 (-0.21,0.04), p=0.161 | 0.97 (0.62,1.52), p=0.909 | 1.40 (1.02,1.90), p=0.036 |
|  | Girls | -12.62 (-18.96, -6.28), p<0.001 | 1.20 (1.01,1.42), p=0.037 | 1.62 (1.19,2.20), p=0.002 | | 0.88 (0.64,1.21), p=0.433 | 1.59 (1.19,2.13), p=0.002 | 0.11 ( 0.00,0.22), p=0.061 | 1.12 (0.78,1.61), p=0.523 | 1.18 (0.91,1.54), p=0.204 |
| **Parent convicted offence** | Basic | -38.41 (-48.60,-28.22), p<0.001 | 1.79 (1.48,2.15), p<0.001 | 1.43 (0.96,2.14), p=0.079 | | 1.38 (0.97,1.97), p=0.074 | 1.61 (1.17,2.22), p=0.004 | 0.05 (-0.09,0.19), p=0.466 | 1.04 (0.64,1.68), p=0.872 | 1.49 (1.10,2.02), p=0.011 |
|  | Adjusted | -16.34 (-25.10, -7.59), p<0.001 | 1.27 (1.04,1.56), p=0.021 | 1.18 (0.77,1.81), p=0.436 | | 1.33 (0.92,1.93), p=0.131 | 1.40 (0.99,1.98), p=0.054 | -0.01 (-0.16,0.13), p=0.849 | 0.83 (0.50,1.38), p=0.472 | 1.28 (0.93,1.77), p=0.134 |
|  | Adjusted complete cases | -11.03 (-18.96, -3.11), p=0.006 | 1.24 (0.96,1.59), p=0.091 | 1.32 (0.75,2.19), p=0.311 | | 1.75 (1.07,2.74), p=0.020 | 1.52 (0.98,2.29), p=0.052 | -0.03 (-0.21,0.14), p=0.711 | 0.79 (0.39,1.44), p=0.478 | 1.22 (0.79,1.82), p=0.350 |
|  | Sex interaction test | p=0.477 | p=0.620 | p=0.945 | | p=0.751 | p=0.662 | p=0.196 | p=0.127 | p=0.595 |
|  | Sex interaction term | -5.64 (-21.25,  9.96), p=0.477 | 1.10 (0.75,1.61), p=0.621 | 0.96 (0.36,2.58), p=0.941 | | 1.14 (0.51,2.51), p=0.751 | 1.15 (0.61,2.20), p=0.665 | 0.19 (-0.10,0.47), p=0.196 | 2.32 (0.76,7.05), p=0.138 | 1.19 (0.63,2.22), p=0.590 |
|  | Boys | -12.76 (-25.30, -0.23), p=0.046 | 1.21 (0.90,1.61), p=0.202 | 1.20 (0.50,2.87), p=0.684 | | 1.28 (0.71,2.31), p=0.411 | 1.41 (0.86,2.31), p=0.169 | -0.09 (-0.30,0.12), p=0.393 | 0.50 (0.18,1.38), p=0.181 | 1.21 (0.72,2.05), p=0.466 |
|  | Girls | -19.84 (-31.24, -8.45), p<0.001 | 1.35 (1.02,1.78), p=0.034 | 1.18 (0.70,2.01), p=0.528 | | 1.37 (0.80,2.35), p=0.255 | 1.42 (0.88,2.28), p=0.148 | 0.05 (-0.14,0.25), p=0.588 | 1.07 (0.59,1.97), p=0.816 | 1.36 (0.90,2.05), p=0.149 |
| **Parental separation** | Basic | -41.05 (-46.63,-35.47), p<0.001 | 1.83 (1.64,2.05), p<0.001 | 1.57 (1.21,2.04), p<0.001 | | 1.27 (1.00,1.62), p=0.054 | 1.67 (1.36,2.05), p<0.001 | 0.07 (-0.02,0.16), p=0.132 | 1.23 (0.95,1.59), p=0.112 | 1.85 (1.53,2.23), p<0.001 |
|  | Adjusted | -17.36 (-22.91,-11.81), p<0.001 | 1.23 (1.08,1.40), p=0.002 | 1.28 (0.96,1.71), p=0.090 | | 1.18 (0.90,1.54), p=0.228 | 1.46 (1.16,1.85), p=0.001 | -0.02 (-0.12,0.08), p=0.731 | 0.99 (0.74,1.32), p=0.931 | 1.56 (1.27,1.93), p<0.001 |
|  | Adjusted complete cases | -13.00 (-17.93, -8.08), p<0.001 | 1.20 (1.02,1.41), p=0.025 | 1.15 (0.80,1.65), p=0.442 | | 0.93 (0.64,1.33), p=0.687 | 1.50 (1.12,2.00), p=0.006 | -0.06 (-0.17,0.05), p=0.270 | 0.99 (0.67,1.43), p=0.969 | 1.57 (1.20,2.05), p<0.001 |
|  | Sex interaction test | p=0.286 | p=0.368 | p=1.000 | | p=0.824 | p=0.842 | p=0.032 | p=0.096 | p=0.853 |
|  | Sex interaction term | 5.29 ( -4.45, 15.04), p=0.286 | 0.90 (0.71,1.14), p=0.365 | 0.99 (0.55,1.76), p=0.968 | | 0.94 (0.57,1.57), p=0.815 | 0.96 (0.63,1.46), p=0.838 | 0.19 ( 0.02,0.36), p=0.032 | 1.62 (0.92,2.85), p=0.093 | 1.04 (0.72,1.50), p=0.846 |
|  | Boys | -20.03 (-28.23,-11.82), p<0.001 | 1.32 (1.11,1.57), p=0.002 | 1.20 (0.68,2.12), p=0.520 | | 1.29 (0.86,1.93), p=0.221 | 1.63 (1.16,2.30), p=0.005 | -0.11 (-0.26,0.03), p=0.124 | 0.73 (0.44,1.22), p=0.227 | 1.66 (1.22,2.26), p=0.001 |
|  | Girls | -15.21 (-22.63, -7.78), p<0.001 | 1.16 (0.96,1.41), p=0.124 | 1.33 (0.95,1.85), p=0.100 | | 1.11 (0.76,1.62), p=0.592 | 1.35 (0.98,1.86), p=0.067 | 0.06 (-0.07,0.19), p=0.383 | 1.18 (0.81,1.71), p=0.386 | 1.53 (1.16,2.03), p=0.003 |

 GCSE - General Certificate of Secondary Education
