# Supplementary material for: Associations of adverse childhood experiences with educational attainment and adolescent health and the role of family and socioeconomic factors: A prospective cohort study in the UK
Source: PLoS Med. 2020 Mar 2;17(3):e1003031. doi: 10.1371/journal.pmed.1003031 (PMC7051040; doi:10.1371/journal.pmed.1003031)
Supplement: S8 Table — The basic model was adjusted for sex, whereas the adjusted model also includes sociodemographic indicators. For convenience, the AOR for the models with a continuous maternal age variable (AORcon) as well as percentage of difference in the AOR are also included. ACE, adverse childhood experience; AOR, adjusted odds ratio; CI, confidence interval. (DOCX) [file pmed.1003031.s013.docx]

*S8 Table: Adjusted odds ratios (AOR), 95% confidence intervals (CIs) and p-values for the association between the ACE measures and educational attainment or health for the sensitivity analysis with maternal age as a categorical variable (less than 20 years, 20–34 years or over 35 years). The basic model was adjusted for sex, whereas the adjusted model also includes sociodemographic indicators. For convenience, the AOR for the models with a continuous maternal age variables (AOR_con_) as well as percentage of difference in AOR are also included.*

| **Adversity** | **Model** | **Analysis 1: Education** | **Analysis 2: Health** | | | | |
| --- | --- | --- | --- | --- | --- | --- | --- |
|  |  | <5 good GCSE  AOR 95% CI, p; AOR_con,_ percentage AOR change | Depression  AOR 95% CI, p; AOR_con_, percentage AOR change | Harmful alcohol use  AOR 95% CI, p; AOR_con,_ percentage AOR change | Illicit drug use AOR 95% CI, p; AOR_con_, percentage AOR change | Obesity  AOR 95% CI, p; AOR_con_, percentage AOR change | Smoking  AOR 95% CI, p; AOR_con_, percentage AOR change |
| **Categorical ACE score** | Basic 1 ACE | 1.41 (1.19,1.67), p<0.001; AOR_con_ 1.38, 2.5% | 1.37 (0.89,2.11), p=0.150; AOR_con_ 1.35, 1.5% | 1.02 (0.73,1.44), p=0.908; AOR_con_ 1.03, -0.8% | 1.43 (1.04,1.97), p=0.028; AOR_con_ 1.45, -1.7% | 1.37 (0.91,2.06), p=0.137; AOR_con_ 1.40, -2.2% | 1.18 (0.90,1.56), p=0.230; AOR_con_ 1.19, -0.3% |
|  | Basic 2-3 ACEs | 1.85 (1.58,2.16), p<0.001; AOR_con_ 1.77, 4.7% | 2.12 (1.43,3.15), p<0.001; AOR_con_ 2.14, -1.0% | 1.31 (0.96,1.77), p=0.084; AOR_con_ 1.32, -0.8% | 1.85 (1.38,2.50), p<0.001; AOR_con_ 1.88, -1.6% | 1.56 (1.06,2.29), p=0.024; AOR_con_ 1.59, -2.1% | 1.69 (1.31,2.19), p<0.001; AOR_con_ 1.70, -0.3% |
|  | Basic 4+ ACEs | 3.18 (2.68,3.78), p<0.001; AOR_con_ 3.18, 0.1% | 3.09 (2.06,4.63), p<0.001; AOR_con_ 3.09, 0.1% | 1.58 (1.13,2.21), p=0.008; AOR_con_ 1.57, 0.4% | 3.22 (2.35,4.43), p<0.001; AOR_con_ 3.35, -3.9% | 1.85 (1.24,2.76), p=0.003; AOR_con_ 1.87, -1.3% | 2.72 (2.08,3.57), p<0.001; AOR_con_ 2.72, 0.3% |
|  | Adjusted 1 ACE | 1.37 (1.14,1.65), p<0.001; AOR_con_ 1.37, 0.1% | 1.34 (0.87,2.08), p=0.181; AOR_con_ 1.29, 4.0% | 1.00 (0.71,1.41), p=0.992; AOR_con_ 0.98, 1.4% | 1.41 (1.02,1.95), p=0.037; AOR_con_ 1.44, -1.9% | 1.33 (0.88,2.02), p=0.178; AOR_con_ 1.38, -3.4% | 1.16 (0.88,1.54), p=0.296; AOR_con_ 1.17, -0.7% |
|  | Adjusted 2-3 ACEs | 1.59 (1.35,1.88), p<0.001; AOR_con_ 1.57, 1.4% | 2.04 (1.36,3.05), p<0.001; AOR_con_ 1.94, 5.0% | 1.26 (0.92,1.73), p=0.147; AOR_con_ 1.21, 4.0% | 1.78 (1.31,2.41), p<0.001; AOR_con_ 1.81, -1.7% | 1.45 (0.97,2.14), p=0.067; AOR_con_ 1.51, -4.3% | 1.62 (1.24,2.11), p<0.001; AOR_con_ 1.63, -0.4% |
|  | Adjusted 4+ ACEs | 2.10 (1.73,2.57), p<0.001; AOR_con_ 2.00, 5.2% | 2.61 (1.68,4.07), p<0.001; AOR_con_ 2.43, 7.4% | 1.45 (1.00,2.10), p=0.048; AOR_con_ 1.36, 6.5% | 2.95 (2.08,4.18), p<0.001; AOR_con_ 3.06, -3.7% | 1.39 (0.89,2.19), p=0.149; AOR_con_ 1.42, -1.9% | 2.33 (1.73,3.14), p<0.001; AOR_con_ 2.26, 2.9% |
| **Physical abuse** | Basic | 1.15 (1.00,1.33), p=0.048; AOR_con_ 1.15, 0.5% | 1.96 (1.49,2.60), p<0.001; AOR_con_ 1.97, -0.2% | 1.30 (1.00,1.69), p=0.046; AOR_con_ 1.28, 1.6% | 1.63 (1.27,2.09), p<0.001; AOR_con_ 1.68, -3.3% | 1.44 (1.08,1.92), p=0.013; AOR_con_ 1.45, -1.1% | 1.82 (1.48,2.23), p<0.001; AOR_con_ 1.80, 0.7% |
|  | Adjusted | 0.94 (0.80,1.10), p=0.445; AOR_con_ 0.91, 3.6% | 1.86 (1.39,2.50), p<0.001; AOR_con_ 1.85, 0.6% | 1.27 (0.97,1.66), p=0.086; AOR_con_ 1.22, 4.3% | 1.55 (1.19,2.01), p=0.001; AOR_con_ 1.58, -2.3% | 1.31 (0.97,1.76), p=0.079; AOR_con_ 1.32, -1.0% | 1.69 (1.36,2.10), p<0.001; AOR_con_ 1.65, 2.6% |
| **Sexual abuse** | Basic | 1.79 (1.39,2.30), p<0.001; AOR_con_ 1.83, -2.1% | 2.25 (1.50,3.37), p<0.001; AOR_con_ 2.28, -1.2% | 1.59 (1.03,2.48), p=0.038; AOR_con_ 1.53, 4.4% | 1.56 (1.04,2.35), p=0.034; AOR_con_ 1.54, 1.4% | 1.70 (1.05,2.74), p=0.031; AOR_con_ 1.70, -0.4% | 1.95 (1.37,2.78), p<0.001; AOR_con_ 1.96, -0.4% |
|  | Adjusted | 1.37 (1.02,1.83), p=0.035; AOR_con_ 1.35, 1.4% | 2.06 (1.34,3.17), p<0.001; AOR_con_ 2.07, -0.5% | 1.54 (0.97,2.43), p=0.065; AOR_con_ 1.46, 5.6% | 1.45 (0.95,2.21), p=0.088; AOR_con_ 1.41, 2.8% | 1.32 (0.80,2.18), p=0.279; AOR_con_ 1.34, -1.2% | 1.66 (1.14,2.41), p=0.008; AOR_con_ 1.60, 3.9% |
| **Emotional abuse** | Basic | 1.35 (1.20,1.52), p<0.001; AOR_con_ 1.36, -0.6% | 1.68 (1.28,2.21), p<0.001; AOR_con_ 1.65, 1.6% | 1.22 (0.95,1.56), p=0.120; AOR_con_ 1.22, 0.0% | 1.78 (1.42,2.23), p<0.001; AOR_con_ 1.85, -3.8% | 1.09 (0.82,1.46), p=0.553; AOR_con_ 1.12, -2.7% | 1.46 (1.19,1.78), p<0.001; AOR_con_ 1.45, 0.1% |
|  | Adjusted | 1.18 (1.02,1.35), p=0.022; AOR_con_ 1.14, 3.6% | 1.53 (1.14,2.04), p=0.004; AOR_con_ 1.42, 7.7% | 1.16 (0.89,1.51), p=0.275; AOR_con_ 1.12, 3.4% | 1.63 (1.28,2.07), p<0.001; AOR_con_ 1.65, -1.1% | 0.96 (0.71,1.31), p=0.803; AOR_con_ 1.00, -3.5% | 1.32 (1.07,1.64), p=0.011; AOR_con_ 1.27, 3.8% |
| **Emotional neglect** | Basic | 2.31 (1.99,2.67), p<0.001; AOR_con_ 2.31, 0.0% | 1.25 (0.94,1.67), p=0.125; AOR_con_ 1.24, 1.3% | 0.96 (0.72,1.28), p=0.775; AOR_con_ 0.96, -0.1% | 1.09 (0.85,1.39), p=0.493; AOR_con_ 1.12, -2.8% | 1.46 (1.10,1.94), p=0.009; AOR_con_ 1.48, -1.4% | 1.14 (0.92,1.42), p=0.230; AOR_con_ 1.17, -2.8% |
|  | Adjusted | 1.91 (1.64,2.22), p<0.001; AOR_con_ 1.90, 0.2% | 1.14 (0.85,1.55), p=0.379; AOR_con_ 1.14, 0.2% | 0.94 (0.69,1.26), p=0.664; AOR_con_ 0.94, -0.7% | 1.05 (0.81,1.35), p=0.710; AOR_con_ 1.09, -4.1% | 1.22 (0.91,1.63), p=0.190; AOR_con_ 1.25, -2.6% | 1.03 (0.82,1.29), p=0.797; AOR_con_ 1.06, -2.8% |
| **Bullying** | Basic | 1.49 (1.33,1.68), p<0.001; AOR_con_ 1.52, -1.7% | 1.66 (1.30,2.12), p<0.001; AOR_con_ 1.65, 0.8% | 1.18 (0.94,1.48), p=0.156; AOR_con_ 1.18, -0.2% | 1.22 (0.99,1.51), p=0.062; AOR_con_ 1.23, -0.4% | 1.28 (0.99,1.65), p=0.058; AOR_con_ 1.27, 0.4% | 1.25 (1.04,1.50), p=0.016; AOR_con_ 1.26, -0.3% |
|  | Adjusted | 1.47 (1.29,1.68), p<0.001; AOR_con_ 1.48, -0.5% | 1.58 (1.23,2.04), p<0.001; AOR_con_ 1.55, 2.1% | 1.13 (0.90,1.43), p=0.292; AOR_con_ 1.13, 0.7% | 1.17 (0.94,1.45), p=0.166; AOR_con_ 1.17, 0.0% | 1.19 (0.92,1.54), p=0.186; AOR_con_ 1.20, -0.3% | 1.18 (0.98,1.43), p=0.081; AOR_con_ 1.18, -0.3% |
| **Violence between parents** | Basic | 1.55 (1.36,1.77), p<0.001; AOR_con_ 1.59, -2.8% | 1.07 (0.79,1.46), p=0.658; AOR_con_ 1.07, 0.6% | 1.26 (0.97,1.64), p=0.087; AOR_con_ 1.24, 1.6% | 1.70 (1.35,2.12), p<0.001; AOR_con_ 1.76, -3.5% | 1.21 (0.90,1.64), p=0.210; AOR_con_ 1.21, 0.3% | 1.58 (1.29,1.94), p<0.001; AOR_con_ 1.57, 0.8% |
|  | Adjusted | 1.18 (1.01,1.37), p=0.032; AOR_con_ 1.14, 3.5% | 0.90 (0.64,1.26), p=0.521; AOR_con_ 0.85, 5.2% | 1.17 (0.89,1.55), p=0.250; AOR_con_ 1.13, 3.8% | 1.53 (1.21,1.95), p<0.001; AOR_con_ 1.57, -2.5% | 1.08 (0.78,1.50), p=0.647; AOR_con_ 1.04, 3.8% | 1.40 (1.13,1.75), p=0.002; AOR_con_ 1.36, 3.5% |
| **Substance use household** | Basic | 1.70 (1.46,1.97), p<0.001; AOR_con_ 1.72, -1.5% | 1.31 (0.91,1.89), p=0.143; AOR_con_ 1.29, 1.9% | 1.67 (1.22,2.29), p=0.001; AOR_con_ 1.62, 3.1% | 2.50 (1.93,3.23), p<0.001; AOR_con_ 2.53, -1.2% | 0.97 (0.64,1.48), p=0.900; AOR_con_ 1.00, -3.1% | 1.85 (1.43,2.40), p<0.001; AOR_con_ 1.80, 3.0% |
|  | Adjusted | 1.10 (0.92,1.32), p=0.300; AOR_con_ 1.07, 2.7% | 1.03 (0.69,1.54), p=0.873; AOR_con_ 0.96, 8.1% | 1.54 (1.09,2.18), p=0.014; AOR_con_ 1.48, 4.2% | 2.17 (1.63,2.88), p<0.001; AOR_con_ 2.14, 1.2% | 0.78 (0.50,1.23), p=0.287; AOR_con_ 0.79, -0.7% | 1.60 (1.20,2.13), p=0.001; AOR_con_ 1.49, 7.4% |
| **Parental mental health problems or suicide** | Basic | 1.54 (1.39,1.71), p<0.001; AOR_con_ 1.57, -1.6% | 1.67 (1.32,2.11), p<0.001; AOR_con_ 1.69, -1.3% | 1.14 (0.92,1.41), p=0.226; AOR_con_ 1.14, 0.4% | 1.49 (1.24,1.79), p<0.001; AOR_con_ 1.53, -2.3% | 1.19 (0.94,1.51), p=0.157; AOR_con_ 1.21, -1.5% | 1.43 (1.20,1.69), p<0.001; AOR_con_ 1.43, -0.2% |
|  | Adjusted | 1.24 (1.10,1.41), p<0.001; AOR_con_ 1.20, 3.7% | 1.54 (1.18,2.00), p=0.001; AOR_con_ 1.44, 6.7% | 1.07 (0.85,1.34), p=0.588; AOR_con_ 0.98, 8.5% | 1.42 (1.16,1.74), p<0.001; AOR_con_ 1.41, 0.4% | 1.07 (0.82,1.39), p=0.630; AOR_con_ 1.08, -1.0% | 1.30 (1.08,1.57), p=0.006; AOR_con_ 1.27, 2.3% |
| **Parent _con_victed offence** | Basic | 1.77 (1.48,2.11), p<0.001; AOR_con_ 1.79, -0.8% | 1.46 (0.96,2.24), p=0.079; AOR_con_ 1.43, 2.1% | 1.42 (0.99,2.05), p=0.059; AOR_con_ 1.38, 2.9% | 1.62 (1.19,2.20), p=0.002; AOR_con_ 1.61, 0.2% | 1.07 (0.68,1.68), p=0.773; AOR_con_ 1.04, 2.7% | 1.56 (1.15,2.11), p=0.004; AOR_con_ 1.49, 4.6% |
|  | Adjusted | 1.33 (1.08,1.63), p=0.007; AOR_con_ 1.27, 4.4% | 1.24 (0.79,1.95), p=0.343; AOR_con_ 1.18, 4.9% | 1.40 (0.95,2.05), p=0.088; AOR_con_ 1.33, 4.9% | 1.45 (1.05,2.00), p=0.026; AOR_con_ 1.40, 3.0% | 0.87 (0.54,1.41), p=0.581; AOR_con_ 0.83, 5.2% | 1.38 (1.00,1.91), p=0.051; AOR_con_ 1.28, 7.7% |
| **Parental separation** | Basic | 1.83 (1.63,2.05), p<0.001; AOR_con_ 1.83, -0.2% | 1.54 (1.19,2.00), p=0.001; AOR_con_ 1.57, -1.7% | 1.27 (1.00,1.62), p=0.054; AOR_con_ 1.27, -0.1% | 1.62 (1.32,2.00), p<0.001; AOR_con_ 1.67, -2.5% | 1.24 (0.94,1.65), p=0.125; AOR_con_ 1.23, 1.1% | 1.86 (1.55,2.23), p<0.001; AOR_con_ 1.85, 0.7% |
|  | Adjusted | 1.30 (1.14,1.49), p<0.001; AOR_con_ 1.23, 5.7% | 1.29 (0.96,1.73), p=0.088; AOR_con_ 1.28, 0.6% | 1.18 (0.90,1.54), p=0.234; AOR_con_ 1.18, 0.0% | 1.44 (1.15,1.81), p=0.001; AOR_con_ 1.46, -1.4% | 1.02 (0.75,1.38), p=0.910; AOR_con_ 0.99, 3.1% | 1.64 (1.34,2.00), p<0.001; AOR_con_ 1.56, 4.6% |
