# Supplementary material for: Associations of adverse childhood experiences with educational attainment and adolescent health and the role of family and socioeconomic factors: A prospective cohort study in the UK
Source: PLoS Med. 2020 Mar 2;17(3):e1003031. doi: 10.1371/journal.pmed.1003031 (PMC7051040; doi:10.1371/journal.pmed.1003031)
Supplement: S9 Table — ACE, adverse childhood experience; AOR, adjusted odds ratio; CI, confidence interval. (DOCX) [file pmed.1003031.s014.docx]

*S9 Table Estimate or Adjusted odds ratios (AOR), 95% confidence intervals and p-values for the interaction between each ACE and parental social class.*

| **Adversity** | **Analysis 1: Education** | | **Analysis 2: Health** | | | | | |
| --- | --- | --- | --- | --- | --- | --- | --- | --- |
|  | GCSE point score | < Five GCSEs | Depression | Harmful alcohol use | Illicit drug use | BMI z-score | Obesity | Smoking |
| Categorical classic ACEs  1 | -3.21 (-18.17, 11.74), p=0.673 | 0.96 (0.62,1.49), p=0.854 | 0.70 (0.18,2.76), p=0.605 | 0.70 (0.24,2.03), p=0.507 | 0.47 (0.18,1.20), p=0.114 | 0.12 (-0.22,0.46), p=0.496 | 0.75 (0.25,2.26), p=0.610 | 0.68 (0.31,1.52), p=0.348 |
| 2-3 | 3.64 (-10.41, 17.70), p=0.611 | 0.82 (0.55,1.22), p=0.329 | 0.99 (0.32,3.11), p=0.990 | 0.77 (0.32,1.83), p=0.552 | 0.51 (0.23,1.11), p=0.090 | -0.01 (-0.33,0.30), p=0.940 | 0.85 (0.32,2.25), p=0.736 | 0.73 (0.37,1.44), p=0.371 |
| 4+ | -1.08 (-15.22, 13.07), p=0.881 | 0.86 (0.59,1.26), p=0.445 | 0.88 (0.29,2.68), p=0.819 | 0.84 (0.36,1.97), p=0.688 | 0.75 (0.35,1.61), p=0.460 | 0.05 (-0.26,0.37), p=0.735 | 0.82 (0.31,2.16), p=0.685 | 0.66 (0.33,1.29), p=0.221 |
| Physical abuse | -1.35 (-14.50, 11.79), p=0.840 | 1.03 (0.75,1.43), p=0.851 | 0.85 (0.44,1.61), p=0.609 | 1.01 (0.55,1.86), p=0.982 | 1.04 (0.62,1.73), p=0.880 | 0.00 (-0.23,0.22), p=0.971 | 1.45 (0.76,2.77), p=0.253 | 0.91 (0.57,1.44), p=0.682 |
| Sexual abuse | 3.21 (-19.32, 25.74), p=0.779 | 0.88 (0.49,1.60), p=0.677 | 0.75 (0.31,1.84), p=0.537 | 0.47 (0.17,1.30), p=0.145 | 0.77 (0.34,1.76), p=0.532 | 0.07 (-0.32,0.46), p=0.727 | 1.22 (0.46,3.27), p=0.692 | 0.77 (0.38,1.56), p=0.466 |
| Emotional abuse | -2.62 (-13.91,  8.67), p=0.649 | 1.06 (0.80,1.40), p=0.692 | 0.97 (0.53,1.79), p=0.933 | 1.07 (0.56,2.05), p=0.828 | 1.00 (0.61,1.66), p=0.988 | -0.03 (-0.25,0.19), p=0.799 | 0.90 (0.45,1.77), p=0.757 | 0.87 (0.54,1.39), p=0.555 |
| Emotional neglect | -0.53 (-11.56, 10.49), p=0.924 | 1.01 (0.75,1.36), p=0.950 | 1.16 (0.59,2.27), p=0.674 | 1.08 (0.56,2.11), p=0.811 | 1.61 (0.97,2.68), p=0.065 | 0.04 (-0.20,0.27), p=0.750 | 1.04 (0.55,1.97), p=0.901 | 1.27 (0.77,2.08), p=0.343 |
| Bullying | -7.39 (-18.38,  3.60), p=0.187 | 1.17 (0.89,1.55), p=0.263 | 0.93 (0.50,1.71), p=0.814 | 0.84 (0.47,1.50), p=0.545 | 0.81 (0.49,1.34), p=0.422 | -0.10 (-0.31,0.11), p=0.342 | 0.74 (0.40,1.39), p=0.350 | 0.86 (0.56,1.31), p=0.485 |
| Violence between parents | -3.43 (-14.46,  7.61), p=0.542 | 1.00 (0.76,1.32), p=0.991 | 1.00 (0.50,1.98), p=0.991 | 1.05 (0.55,1.99), p=0.888 | 1.20 (0.74,1.97), p=0.458 | 0.00 (-0.22,0.23), p=0.984 | 0.86 (0.44,1.71), p=0.676 | 0.78 (0.49,1.23), p=0.279 |
| Substance household | -4.00 (-16.78,  8.78), p=0.539 | 0.95 (0.69,1.30), p=0.738 | 1.17 (0.56,2.45), p=0.677 | 0.80 (0.40,1.59), p=0.528 | 0.89 (0.51,1.57), p=0.686 | 0.00 (-0.27,0.26), p=0.984 | 1.13 (0.50,2.57), p=0.772 | 0.77 (0.46,1.29), p=0.322 |
| Mental health problems or suicide | -7.16 (-16.58,  2.27), p=0.136 | 1.05 (0.83,1.34), p=0.678 | 1.03 (0.57,1.87), p=0.919 | 1.05 (0.62,1.79), p=0.853 | 1.08 (0.69,1.70), p=0.731 | -0.01 (-0.20,0.18), p=0.906 | 0.92 (0.51,1.63), p=0.769 | 0.84 (0.56,1.24), p=0.375 |
| Parent convicted offence | -9.54 (-24.07,  5.00), p=0.198 | 0.96 (0.66,1.40), p=0.838 | 0.95 (0.40,2.23), p=0.902 | 0.68 (0.30,1.57), p=0.365 | 0.96 (0.49,1.87), p=0.905 | 0.00 (-0.31,0.32), p=0.977 | 0.92 (0.35,2.42), p=0.872 | 0.84 (0.45,1.56), p=0.578 |
| Parental separation | 1.51 ( -8.40, 11.43), p=0.764 | 0.85 (0.67,1.09), p=0.208 | 1.06 (0.57,1.97), p=0.852 | 1.13 (0.65,1.98), p=0.667 | 1.22 (0.72,2.04), p=0.460 | 0.01 (-0.20,0.22), p=0.933 | 1.01 (0.54,1.90), p=0.964 | 0.95 (0.63,1.43), p=0.805 |

 GCSE - General Certificate of Secondary Education
