# Supplementary material for: Associations of adverse childhood experiences with educational attainment and adolescent health and the role of family and socioeconomic factors: A prospective cohort study in the UK
Source: PLoS Med. 2020 Mar 2;17(3):e1003031. doi: 10.1371/journal.pmed.1003031 (PMC7051040; doi:10.1371/journal.pmed.1003031)
Supplement: S10 Table — ACE, adverse childhood experience; AOR, adjusted odds ratio; CI, confidence interval. (DOCX) [file pmed.1003031.s015.docx]

*S10 Table Estimate or Adjusted odds ratios (AOR), 95% confidence intervals and p-values for the interaction between each ACE and maternal education.*

| **Adversity** | **Analysis 1: education** | | | **Analysis 2: health** | | | | | |
| --- | --- | --- | --- | --- | --- | --- | --- | --- | --- |
|  | GCSE point score | Less than five GCSEs | Depression | | Harmful alcohol use | Illicit drug use | BMI z-score | Obesity | Smoking |
| Categorical classic ACEs    1 | -7.97 (-24.63, 8.68), p=0.348 | 0.75 (0.46,1.22), p=0.245 | 0.58 (0.17,1.96), p=0.384 | | 0.88 (0.31,2.47), p=0.806 | 1.06 (0.30, 3.77), p=0.925 | -0.02 (-0.38,0.34), p=0.901 | 0.61 (0.21,1.74), p=0.352 | 0.50 (0.22,1.11), p=0.088 |
| 2-3 | -20.22 (-35.63, -4.80), p=0.010 | 0.65 (0.42,1.01), p=0.055 | 0.52 (0.18,1.53), p=0.237 | | 0.63 (0.25,1.54), p=0.309 | 1.20 (0.39, 3.74), p=0.752 | -0.08 (-0.40,0.23), p=0.606 | 0.52 (0.20,1.35), p=0.180 | 0.56 (0.28,1.12), p=0.102 |
| 4+ | -11.83 (-29.19, 5.54), p=0.182 | 0.88 (0.55,1.42), p=0.603 | 0.67 (0.21,2.07), p=0.484 | | 0.63 (0.22,1.80), p=0.389 | 1.17 (0.35, 3.95), p=0.802 | 0.05 (-0.33,0.42), p=0.808 | 0.64 (0.22,1.81), p=0.399 | 0.52 (0.24,1.12), p=0.096 |
| Physical abuse | 9.82 ( -2.50, 22.15), p=0.118 | 1.18 (0.87,1.61), p=0.292 | 0.79 (0.37,1.65), p=0.523 | | 0.96 (0.51,1.80), p=0.906 | 0.85 (0.49, 1.49), p=0.567 | 0.12 (-0.13,0.37), p=0.343 | 0.82 (0.42,1.60), p=0.553 | 0.99 (0.60,1.64), p=0.982 |
| Sexual abuse | 13.53 ( -9.62, 36.67), p=0.251 | 1.32 (0.73,2.39), p=0.359 | 1.34 (0.55,3.27), p=0.517 | | 0.66 (0.25,1.76), p=0.404 | 0.81 (0.32, 2.05), p=0.655 | 0.04 (-0.36,0.45), p=0.842 | 0.83 (0.29,2.37), p=0.730 | 0.70 (0.32,1.54), p=0.379 |
| Emotional abuse | 12.56 (1.34, 23.78), p=0.028 | 1.33 (1.00,1.78), p=0.050 | 1.12 (0.58,2.19), p=0.732 | | 0.81 (0.43,1.51), p=0.499 | 0.78 (0.44, 1.36), p=0.382 | -0.16 (-0.41,0.08), p=0.198 | 0.58 (0.29,1.18), p=0.134 | 0.70 (0.43,1.14), p=0.150 |
| Emotional neglect | 3.59 ( -7.64, 14.82), p=0.529 | 1.01 (0.75,1.36), p=0.941 | 0.66 (0.33,1.32), p=0.240 | | 0.66 (0.33,1.33), p=0.248 | 1.21 (0.68, 2.16), p=0.514 | 0.12 (-0.11,0.35), p=0.309 | 1.01 (0.53,1.91), p=0.979 | 1.12 (0.69,1.83), p=0.650 |
| Bullying | 0.44 (-10.81, 11.68), p=0.939 | 0.95 (0.72,1.26), p=0.724 | 0.81 (0.43,1.54), p=0.519 | | 0.90 (0.50,1.62), p=0.725 | 0.69 (0.39, 1.22), p=0.202 | 0.03 (-0.18,0.25), p=0.764 | 0.93 (0.51,1.70), p=0.807 | 0.84 (0.55,1.31), p=0.447 |
| Violence between parents | 6.18 (-5.58, 17.93), p=0.302 | 1.16 (0.87,1.55), p=0.302 | 0.87 (0.41,1.85), p=0.716 | | 0.68 (0.33,1.37), p=0.276 | 0.93 (0.54, 1.62), p=0.809 | -0.01 (-0.26,0.23), p=0.929 | 1.07 (0.54,2.09), p=0.852 | 0.88 (0.54,1.41), p=0.589 |
| Substance household | 3.65 (-10.26, 17.57), p=0.606 | 1.20 (0.85,1.69), p=0.308 | 0.97 (0.39,2.45), p=0.956 | | 0.88 (0.40,1.95), p=0.756 | 0.98 (0.50, 1.95), p=0.960 | 0.03 (-0.28,0.35), p=0.829 | 1.09 (0.42,2.80), p=0.865 | 0.79 (0.43,1.45), p=0.441 |
| Mental health problems or suicide | -0.38 (-9.46, 8.69), p=0.934 | 1.09 (0.86,1.38), p=0.458 | 0.82 (0.45,1.52), p=0.534 | | 0.94 (0.55,1.62), p=0.836 | 0.83 (0.51, 1.35), p=0.449 | 0.01 (-0.18,0.21), p=0.918 | 0.97 (0.56,1.69), p=0.924 | 0.73 (0.47,1.12), p=0.145 |
| Parent convicted offence | 11.04 (-5.03, 27.10), p=0.178 | 1.38 (0.93,2.05), p=0.104 | 0.97 (0.37,2.54), p=0.949 | | 0.76 (0.30,1.95), p=0.572 | 0.66 (0.30, 1.45), p=0.305 | -0.06 (-0.39,0.26), p=0.703 | 0.84 (0.30,2.33), p=0.732 | 0.57 (0.29,1.15), p=0.117 |
| Parental separation | 9.82 (-0.71, 20.34), p=0.067 | 1.25 (0.98,1.61), p=0.077 | 0.90 (0.47,1.71), p=0.738 | | 0.99 (0.57,1.71), p=0.962 | 1.10 (0.65, 1.84), p=0.726 | -0.07 (-0.29,0.15), p=0.525 | 0.88 (0.49,1.59), p=0.677 | 0.85 (0.56,1.30), p=0.462 |

 GCSE - General Certificate of Secondary Education
